# Hydrogen peroxide induced by nerve injury promotes axon regeneration via connective tissue growth factor

**DOI:** 10.1186/s40478-022-01495-5

**Published:** 2022-12-25

**Authors:** Samuele Negro, Fabio Lauria, Marco Stazi, Toma Tebaldi, Giorgia D’Este, Marco Pirazzini, Aram Megighian, Francesca Lessi, Chiara M. Mazzanti, Gabriele Sales, Chiara Romualdi, Silvia Fillo, Florigio Lista, James N. Sleigh, Andrew P. Tosolini, Giampietro Schiavo, Gabriella Viero, Michela Rigoni

**Affiliations:** 1 Department of Biomedical Sciences, University of Padua, 35131 Padua, Italy; 2 U.O.C. Clinica Neurologica, Azienda Ospedale, University of Padua, 35128 Padua, Italy; 3 Institute of Biophysics, CNR Unit at Trento, 38123 Povo, Italy; 4 Department of Cellular, Computational and Integrative Biology (CIBIO), University of Trento, 38123 Povo, Italy; 5 Section of Hematology, Department of Internal Medicine, Yale Comprehensive Cancer Center, Yale University School of Medicine, New Haven, CT 06520 USA; 6 Myology Center (CIR-Myo), University of Padua, 35129 Padua, Italy; 7 Padua Neuroscience Center, University of Padua, 35131 Padua, Italy; 8 Laboratory of Genomics, Pisa Science Foundation, 56017 San Giuliano Terme, Italy; 9 Department of Biology, University of Padua, 35131 Padua, Italy; 10 Center of Medical and Veterinary Research of the Ministry of Defence, 00184 Rome, Italy; 11 Department of Neuromuscular Diseases, Queen Square Institute of Neurology, University College London, London, WC1N 3BG UK; 12 UCL Queen Square Motor Neuron Disease Centre, University College London, London, WC1N 3BG UK; 13 UK Dementia Research Institute, University College London, London, WC1E 6BT UK

**Keywords:** Connective tissue growth factor, Hydrogen peroxide, Nerve regeneration, Neuromuscular junction, Schwann cells, Yes-associated protein

## Abstract

Regeneration of the neuromuscular junction (NMJ) leverages on extensive exchange of factors released from motor axon terminals (MATs), muscle fibers and perisynaptic Schwann cells (PSCs), among which hydrogen peroxide (H_2_O_2_) is a major pro-regenerative signal. To identify critical determinants of NMJ remodeling in response to injury, we performed temporal transcriptional profiling of NMJs from 2 month-old mice during MAT degeneration/regeneration, and cross-referenced the differentially expressed genes with those elicited by H_2_O_2_ in SCs. We identified an enrichment in extracellular matrix (ECM) transcripts, including *Connective Tissue Growth Factor* (*Ctgf*), which is usually expressed during development. We discovered that Ctgf levels are increased in a Yes-associated protein (YAP)-dependent fashion in response to rapid, local H_2_O_2_ signaling generated by stressed mitochondria in the injured sciatic nerve, a finding highlighting the importance of signals triggered by mechanical force to motor nerve repair. Through sequestration of Ctgf or inactivation of H_2_O_2_, we delayed the recovery of neuromuscular function by impairing SC migration and, in turn, axon-oriented re-growth. These data indicate that H_2_O_2_ and its downstream effector Ctgf are pro-regenerative factors that enable axonal growth, and reveal a striking ECM remodeling process during nerve regeneration upon local H_2_O_2_ signaling. Our study identifies key transcriptomic changes at the regenerating NMJ, providing a rich source of pro-regenerative factors with potential for alleviating the consequences of peripheral nerve injuries.

**Supplementary Information:**

The online version contains supplementary material available at 10.1186/s40478-022-01495-5.

## Background

Peripheral nerves possess the capacity to regenerate after injury due to intrinsic properties of motor neurons (MNs) and a permissive local environment [1–4]. A complex and finely coordinated nerve injury response is orchestrated by a sequence of transcriptional events that involve the expression of specific sets of pro-survival and pro-regenerative genes [5], whose molecular triggers and mechanisms of action are only partially understood despite intense research. Regeneration of the neuromuscular junction (NMJ), which is the specialized synapse essential for motor function and survival, is the result of an intense cross talk between the motor axon terminal (MAT), perisynaptic Schwann cells (PSCs), muscle fibers, and the basal lamina of the synaptic cleft [1, 6, 7]. This is the best characterized synapse due to its relative simplicity and experimental accessibility, which have facilitated the identification of molecules and mechanisms regulating its formation, maturation, maintenance, function, and regeneration [6–10]. The NMJ is a pathological target of several neurodegenerative diseases where cellular impairment begins prior to the appearance of clinical signs of disease [11–14]. Therefore, identification of additional factors and mechanisms driving synaptic dynamics and remodeling in response to injury holds great promise for a better understanding of the system, and the design of therapeutics able to counteract denervation and promote improved functional recovery.

As a starting point of the study, we unravelled the transcriptional changes that occur at the NMJ upon a rapidly reversible injury to the MAT. To do so, we choose to profile the transcriptome of NMJs in the mouse soleus: indeed, slow-twitch muscles (like the soleus) display a higher degree of plasticity and remodelling capability than fast-twitch muscles (e.g. the EDL) in response to denervation, and are less vulnerable to aging and neurodegenerative conditions [15–17]. We determined the transcriptional profiling of soleus NMJs during the time-course of nerve terminal degeneration and regeneration induced by the pore-forming toxin α-Latrotoxin (α-LTx). This neurotoxin specifically targets the MAT causing an acute, calcium-dependent degeneration that is fully reversible, with complete recovery within a few days in mice [18–20]. The process of MAT degeneration/regeneration by α-LTx is highly reproducible and can be temporally assessed by imaging the progressive disappearance/reappearance of pre-synaptic markers, and through quantitative electrophysiological analyses of neurotransmission [20, 21]. Upon damage, neurons produce hydrogen peroxide (H_2_O_2_), which acts as a major activator of Schwann cells (SCs) both in vivo and in vitro; this manifests as profound morphological changes that allow the clearing of nerve debris by phagocytosis, and the engagement of the pro-regenerative ERK pathway through phosphorylation [20, 22–24]. Therefore, to identify genes most critical to regeneration of the NMJ, we intersected the differentially expressed genes (DEGs) caused by α-LTx with our previously published data set of genes activated by H_2_O_2_ in SCs [23].

Among the top common hits, we identified the mRNA encoding the matricellular protein Connective Tissue Growth Factor (Ctgf), a component of the extracellular matrix (ECM) endowed with signaling function [25]. Ctgf is highly expressed during development and in dynamic processes, such as wound healing and cancer. Given that the role of Ctgf in nerve regeneration in mice had not been previously addressed, we investigated its expression, localization, transcriptional regulation and contribution to recovery after toxin- and injury-induced NMJ damage.

## Methods

### Ethical statement

Eight to ten week-old CD1 mice were employed for laser capture microdissection (LCM) and transcriptional profiling, and for electrophysiological recordings. C57BL/6 mice expressing cytosolic GFP under the *plp* promoter [26] were kindly provided by Dr. W.B. Macklin (Aurora, Colorado), with the help of Dr. T. Misgeld (Munchen, Germany), and used for imaging. The *proteolipid protein* (*plp*) gene promoter drives the expression of one of the major components of myelin predominantly in oligodendrocytes, SCs and the enteric glia of the gut, both in embryonic and postnatal tissues. Originally created to label oligodendrocytes in the central nervous system, the *plp*-GFP mice employed in the present study express cytoplasmic GFP in both myelinating and non-myelinating SCs of the peripheral nerves [26]. Mice expressing the Tomato protein specifically in ChAT (choline acetyltransferase)-positive neurons were obtained by crossing the C57BL/6 ChAT-Cre knock-in mice line with C57BL/6 Rosa26.tdTomato mice (Jackson Laboratories). C57BL/6 mice (8–12 weeks) were also used. Mice were maintained under a 12-h light/12-h dark cycle, and kept under constant temperature. Water and food were available ad libitum, and mice were fed with regular chow. All surgical procedures were performed under general anaesthesia (a cocktail of xilazine (48 mg/kg) and zoletil (16 mg/kg) via intraperitoneal injections or via isoflurane as reported in [27]). Paralysis was restricted to one hind limb, and did not impair food or water intake.

All experimental procedures involving animals and their care comply with the ARRIVE guidelines. Procedures carried out in Italy were approved by the ethical committee and by the animal welfare coordinator of the OPBA from the University of Padua. All procedures are specified in the projects approved by the Italian Ministry of Health, Ufficio VI (authorisation numbers: 359/2015 PR; 81/2017 PR; 521/2018 PR; 439/2019 PR) and were conducted in accordance with National laws and policies (D.L. n. 26, March 14, 2014), following the guidelines established by the European Community Council Directive (2010/63/EU) for the care and use of animals for scientific purposes. Animals were handled by specialised personnel under the control of inspectors from the Veterinary Service of the Local Sanitary Service (ASL 16-Padua), who are the local officers of the Ministry of Health.

Animal experiments in the United Kingdom were conducted in accordance with the European Community Council Directive of 24 November 1986 (86/609/EEC), under license from the UK Home Office in accordance with the Animals (Scientific Procedures) Act 1986, and were approved by the UCL Institute of Neurology Ethical Review Committee.

### Toxins and antibodies

α-LTx (LSP-130) and µ-conotoxin GIIIB (C-270) were purchased from Alomone. α-LTx purity was checked by SDS–PAGE, and its neurotoxicity by ex vivo mouse nerve-hemidiaphragm preparations, as previously described [28]. BoNT/A (Xeomin) was from Merz. All other reagents were from Sigma unless stated otherwise.

Antibodies and fluorescent conjugates with relative dilutions: α-BTx AlexaFluor555 (B35451 Thermo Fisher, 1:200), anti-Ctgf neutralizing antibody (70R-CR023 Fitzgerald, 2 µg/40 µl), anti-Ctgf for immunostaining (ab6992 Abcam, 1:200), anti-S100 (Z0311 Dako, 1:400), anti-GAP43 (ab75810 Abcam, 1:200), anti-NF (ab4680 Abcam, 1:800), anti-VAMP1 [29], anti-SNAP25 (ab24737 Abcam, 1:200), anti-SNAP-25 BoNT/A-cleaved [30], anti-syntaxin 1A/1B [31], anti-YAP (13008S, Cell Signaling, 1:200). Secondary AlexaFluor-conjugated antibodies (1:200) were from Thermo Fisher. A list of antibodies and the relative description is provided as Additional file 9.

### Sample preparation and RNA extraction

Upon isoflurane anaesthetization, CD1 male mice of ≈ 20 g were injected in the hind limb with α-LTx (5 µg/kg in 15 µl physiological saline, 0.9% NaCl + 0.2% gelatine) or vehicle (15 µl saline). A few minutes before muscle collection, a local injection of fluorescent α-BTx was performed to visualize NMJs. At different time points (0, 4, 16, 72 and 168 h), treated mice (5 mice/group) were sacrificed by anaesthetic overdose (a cocktail of xilazine (48 mg/kg) and zoletil (16 mg/kg) via intraperitoneal injections) followed by cervical dislocation; soleus muscles were then rapidly collected, and frozen in liquid nitrogen-cooled isopentane. Cryo-sections (7–10 µm thick) were transferred to UV-treated microscope glass slides. Microdissection was performed with a PALM RoboMover automatic laser microdissector (Carl Zeiss, Oberkochen, Germany). One hundred NMJs/sample were collected by LCM within 30 min, to preserve RNA integrity, and pooled. Total RNA was isolated by incubation with 50 µl lysis buffer PKD (Qiagen) and 10 µl proteinase K (Promega) at 55 °C overnight, with the sample upside down. The next day, samples were centrifuged for 10 min at 10,000 rpm, and RNA extracted using the Maxwell® 16 LEV RNA FFPE Purification Kit with automated system Maxwell 16 (Promega). RNA extraction was performed following the manufacturer’s protocol starting from the DNase treatment step.

### Library preparation

cDNA was obtained using the SMARTer Universal Low Input RNA kit (Clontech Laboratories), according to manufacturer’s instructions. Libraries for RNA-Seq were obtained using the Nextera XT kit (Illumina) according to the manufacturer’s guidelines. RNA sequencing was performed with NextSeq 500 (Illumina), loading a maximum of six, pooling libraries for each cartridge NextSeq High Output (300 cycles).

#### Computational analysis of sequencing data

One hundred bp paired-end reads were processed by removing Illumina Nextera adapters using Trimmomatic (v0.36). Processed reads were aligned to the mouse genome (GRCm38.p6) with STAR (v020201), using the Gencode M17 gene annotation, based on ENSEMBL release 92. Read duplicates were removed with Picard Tools MarkDuplicates (v2.18.9). All programs were used with default settings unless otherwise specified.

**Fig. 1:**
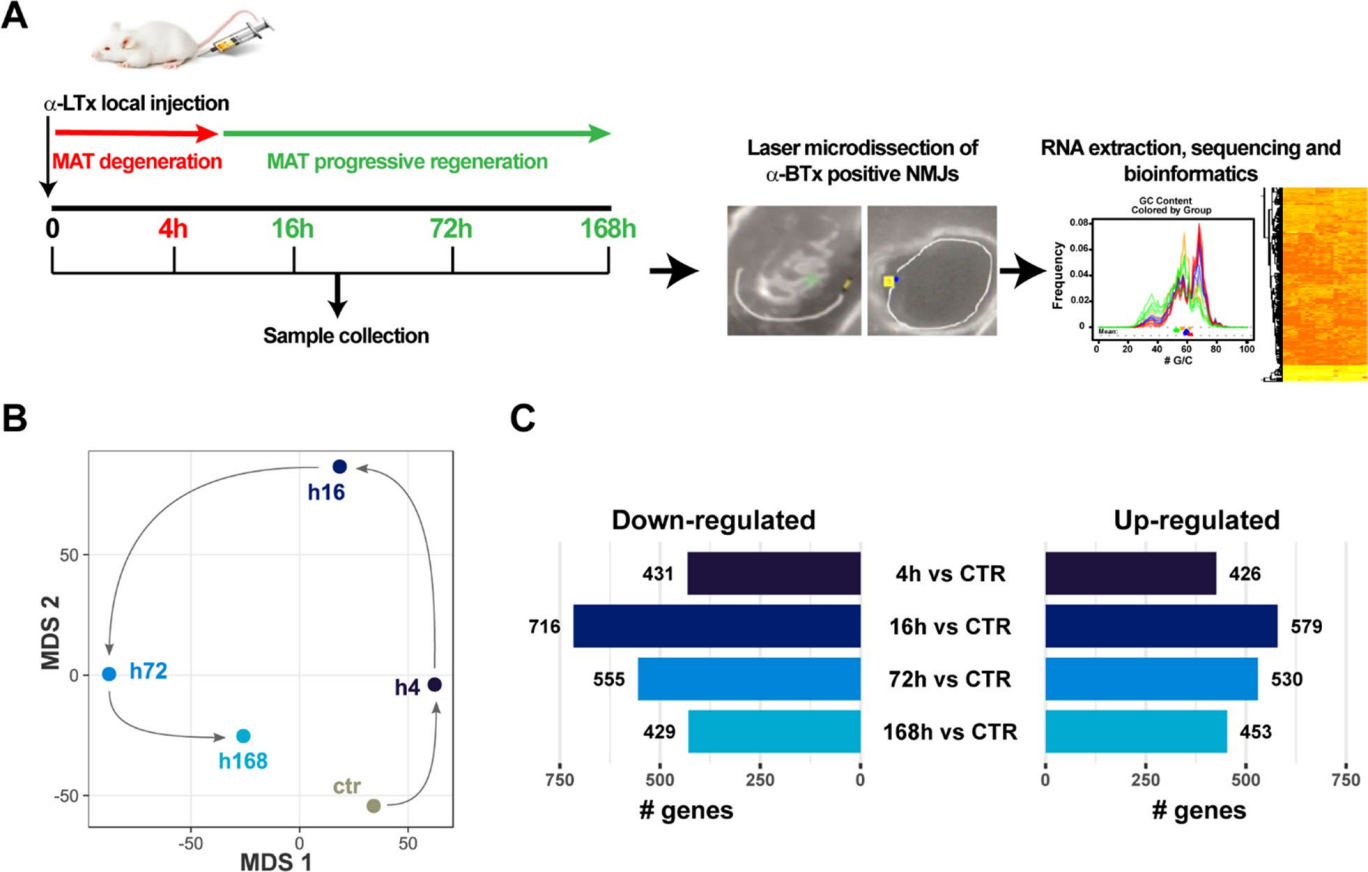
NMJ transcriptional profiling during MAT degeneration and regeneration. **A** Project workflow. CD1 mice were locally injected with α-LTx in the hind limb, and soleus muscles collected 4, 16, 72 and 168 h post-treatment. Contralateral muscles received saline only. Before muscle collection, fluorescent α-BTx was locally injected to label the post-synaptic compartment. Muscles were frozen, cryosliced, and the NMJs collected by laser microdissection and pooled. RNA was extracted, retro-transcribed, amplified and sequenced. **B** Multi-dimensional scaling analysis (MDS) based on the average expression values of all the expressed genes for each time point. Arrows highlight the circular trajectory, which is consistent with MAT degeneration and regeneration. **C** Differentially expressed genes (DEGs) down- (left) and up-regulated (right) with respect to controls at the four time points analyzed (4, 16, 72 and 168 h)

Multidimensional scaling (MDS) was performed with the *cmdscale* function of the "stats" package in R [32]. The analysis was based on the scaled expression values of 10,956 genes, determined at each time point, averaging the signals from replicates. The Euclidean distance was used as the distance metric in the analysis in Fig. 1B.

Gene expression levels were normalized among replicates using the TMM method implemented in the edgeR Bioconductor package [33]. Differentially expressed genes were detected using edgeR with a double threshold on the log_2_-fold change (absolute value > 0.75), and the correspondent statistical significance (*P* < 0.05). Functional annotation of gene lists and enrichment analysis with Gene Ontology terms and KEGG pathways were performed with the clusterProfiler Bioconductor package.

Differentially-expressed genes resulting from H_2_O_2_ treatment in *R. norvegicus* were from [23]. Paralogous *M. musculus* genes were retrieved from ENSEMBL (release 92). Common up- and down-regulated genes in the two data sets were defined as the intersection of genes up- or down-regulated in at least one of the four time points of α-LTx treatment (4, 16, 72 and 168 h), and in at least one of the two time points upon H_2_O_2_ treatment (20 and 40 min).

#### Droplet digital PCR

Droplet digital PCR (ddPCR) was carried out using the ddPCRTM Supermix for Probes (No dUTP), the QX200TM Droplet Generator, the QX200 Droplet Reader, the C1000 TouchTM Thermal Cycler and the PX1TM PCR Plate Sealer (BIO-RAD, Hercules, California, USA) following the manufacturer’s instructions. Reactions were performed in triplicate in a 96 well plate using 10 μL/reaction of 2 × ddPCR Supermix for Probes (No dUTP), 1 μL/reaction of 20 × target primers/probe (FAM or HEX, BIO-RAD), 1 μL/reaction 20x reference primers/probe (FAM or HEX, BIO-RAD), 3 μL cDNA and 5 μL H_2_O. Detection of Ctgf and Gapdh by ddPCR was performed using the following PrimePCR™ ddPCR™ Expression Probe Assay designed by BIO-RAD: CTGF-FAM (ID: qMmuCEP0053713) and GAPDH-HEX (ID: dMmuCPE5195283, BIO-RAD). All steps used a ramp rate of 2˚C/s. Results were analyzed in the QX200 Droplet Reader, the RNA targets were quantified using the QuantaSoftTM Software (BIO-RAD), and results were normalized to the control.

### Electrophysiological recordings of evoked junctional potentials (EJPs)

EJPs were intracellularly recorded in vitro from single soleus muscle fibers following NMJ poisoning by α-LTx. Anaesthetized mice were locally injected with α-LTx (5 μg/kg in 15 μL of 0.9% NaCl, 0.2% gelatin) in the hind limb [20, 21]. In a group of animals, after the local injection of the toxin, 2 µg of anti-Ctgf neutralizing antibody (in 40 µl physiological solution plus 0.2% gelatine) were intraperitoneally injected to induce the biochemical knockout of the molecule [21, 34], which begins rapidly after antibody injection and lasts for a long time, as the half-life of murine IgG antibodies exceeds 11 days [35]. 96 h later, when 50% neurotransmission blockade by the toxin is achieved (a value very suitable for assessing the impact of a treatment on nerve recovery of function), mice were sacrificed by cervical dislocation, and soleus muscles quickly excised and pinned to a Sylgard-coated petri dish (Sylgard 184, Down Corning USA). Recordings were performed in oxygenated Krebs–Ringer solution using intracellular glass microelectrodes (1.5 mm outer diameter, 1.0 mm inner diameter, 15–20 MΩ tip resistance; GB150TF, Science Products GmbH Germany), filled with a 1:2 solution of 3 M KCl and 3 M CH_3_COOK. Evoked neurotransmitter release was recorded in current-clamp mode, and resting membrane potential was adjusted with current injection to -70 mV. EJPs were elicited by supramaximal nerve stimulation at 0.5 Hz using a suction glass microelectrode (GB150TF, Science Products GmbH Germany) connected to a S88 stimulator (Grass, USA). Muscle fiber contraction during intracellular recordings was blocked by adding 1 μM μ-conotoxin. Intracellularly recorded signals were amplified with an intracellular amplifier (BA-01X, NPI, Germany), digitized using a digital A/C interface (NI PCI-6221, National Instruments, USA), and then fed to a computer for both on-line visualization and off-line analysis using appropriate software (WinEDR, Strathclyde University; pClamp, Axon, USA). Stored data were analyzed off-line using the software pClamp (Axon, USA).

### Sciatic nerve compression/transection

The sciatic nerve was exposed at the level of the sciatic notch under general anaesthesia without damaging the gluteal musculature. Nerve compression (crush) was performed using haemostatic forceps, pre-dipped in powdered charcoal, to mark the crush site, by pinching the nerve 0.5 cm from the hip insertion for 20 s at the 3rd click of the haemostatic forceps. Transection of the sciatic nerve was performed using surgical scissor, leaving the edge juxtaposed [36]. The gluteal musculature was re-opposed and the skin sutured using 6–0 braided silk, non-absorbable sutures (ETHLCON2 biological instruments, 8697).

After surgery, mice were intraperitoneally injected once a week with anti-Ctgf antibodies (2 µg in 40 µl physiological solution plus 0.2% gelatine) or vehicle [34, 37], and electrophysiological measurements (in the case of crush) and immunofluorescence (crush and cut) were performed at different time points after injury.

In selected experiments, after sciatic nerve exposure, but before nerve compression, catalase (C1345, Sigma Aldrich) was intra-sciatically injected (1 mM in physiological solution, 2 μl injection volume) using a pulled graduated glass micropipette, gently inserted in the medial area of the sciatic nerve under the perineurium [38].

### Electrophysiological recordings of compound muscle action potentials (CMAPs)

CMAPs were recorded from mice 18 or 28 days following sciatic nerve crush [37, 39, 40]. The 28 d time-point corresponds to 50–60% neurotransmission recovery following nerve compression, a value that is very suitable for assessing the impact of a treatment on nerve recovery of function [37]. Upon general anaesthesia, the sciatic nerve was exposed at the sciatic notch, and a small piece of parafilm was slid under the nerve, which was kept moist with phosphate buffered saline (PBS). A pair of stimulating needle electrodes (Grass, USA) were then advanced until gently touching the exposed sciatic nerve, above the site of crush lesion, using a mechanical micromanipulator (MM33, FST, Germany). A pair of electromyography needle electrodes (Grass, USA) were used for electromyographic recording of gastrocnemius muscle fibre activity. The recording needle electrode was inserted halfway into the gastrocnemius muscle, while the indifferent needle electrode was inserted in the distal tendon of the muscle. CMAPs were recorded following supramaximal stimulation of the sciatic nerve at 0.5 Hz (0.4 ms stimulus duration) using a stimulator (S88, Grass, USA) via a stimulus isolation unit (SIU5, Grass, USA) in a capacitance coupling mode. To reach supramaximal stimuli (5–15 mV for controls, up to 50 mV after nerve damage), the sciatic nerve was stimulated with increasingly intense stimuli until the CMAP value ceased to increase. Recorded signals were amplified by an extracellular amplifier (P6 Grass, USA), digitized using a digital A/C interface (National Instruments, USA), and then fed to a computer for both on-line visualization and off-line analysis using appropriate software (WinEDR, Strathclyde University; pClamp, Axon, USA). Stored data were analyzed off-line using pClamp software (Axon, USA).

### Immunohistochemistry

Soleus muscles were dissected at different time points after local administration of α-LTx, or 21 days after BoNT/A (0,5 U in 25 µl physiological solution plus 0.2% gelatin), w/wo treatment with anti-Ctgf neutralizing antibodies, then fixed in 4% PFA in PBS for 30 min at RT, and quenched in 0.24% NH_4_Cl PBS for 20 min. After permeabilization and 2 h saturation in blocking solution (15% goat serum, 2% BSA, 0.25% gelatine, 0.20% glycine, 0.5% Triton-X100 in PBS), samples were incubated with primary antibody against syntaxin 1A/1B for 72 h in blocking solution at 4 °C. Muscles were then washed, and incubated with secondary antibodies and α-BTx AlexaFluor-555 to stain post-synaptic acetylcholine receptors (AChRs). Images were collected with a Leica SP5 confocal microscope equipped with a 40 × and 63 × HCX PL APO NA 1.4 oil immersion objective. Laser excitation line, power intensity and emission range were chosen according to each fluorophore in different samples to minimize bleed-through. Orthogonal projection and 3D analysis were performed with ImageJ software (*Orthogonal views* command in *Image-Stacks* section and 3D viewer plugin). The orthogonal projection method was used with a stack to display the XZ and YZ planes at a given point in the 3D image.

Pseudocolor image analysis: the 8 bit image format, acquired by confocal microscope, is associated with a signal intensity gradient (LUT) in grayscale, with pixel intensity values ranging from highest to lowest intensity. The grayscale image is converted in pseudocolors by Fiji imaging software, by assigning colors to the gray levels (pseudocolor Fire). The pseudocolor scale is added in the corresponding figure. White corresponds to the highest intensity, black to the lowest. This colored image, when displayed, allows an easier identification of certain features by the observer.

Sciatic nerves were isolated at different time points following crush or cut, w/wo treatment with anti-Ctgf neutralizing antibodies, fixed in 4% PFA in PBS for 30 min, sucrose-cryoprotected overnight, and embedded in OCT. Samples were slowly frozen in isopentane cooled with liquid nitrogen vapours, and cryo-sliced (20 μm thickness) using a Leica CM1520 cryostat. Slices were processed for immunostaining as described in [37]. In some experiments, whole mount staining of the nerve was performed as described in [41].

### Imaging of H_2_O_2_ in the murine sciatic nerve

Live imaging experiments were performed in C57BL/6 mice following the protocol described in [27]. Briefly, after anaesthesia was initiated and maintained using isoflurane, the fur on the dorsal hind limb (ankle to hip) was shaved off, and mice were placed on a heat-pad for the duration of surgery. An incision was made in the trochanteric region and the skin covering the entire surface was removed, along with the biceps femoris muscle to expose the underlying sciatic nerve. The connective tissue underneath the sciatic nerve was gently disrupted using curved forceps to enable the placement of a small piece of parafilm aiding the subsequent imaging. Once the sciatic nerve was exposed, 1 mM PF6-AM (A14086 AdooQ Bioscience) in 2 μl saline was injected into the nerve. Peroxyfluor-6 acetoxymethyl ester (PF6-AM) is a chemoselective fluorescent indicator for H_2_O_2_. The molecule features a boronate chemical switch that allows for selective detection of H_2_O_2_ over other ROS, combined with acetoxymethylester (AM) protected phenol and carboxylic acid groups for enhanced cellular retention and sensitivity [42]. The anaesthetised mouse was then transferred to an inverted LSM780 laser scanning microscope (Zeiss) equipped within an environmental chamber pre-warmed and maintained at 37 °C. Using a 10x, Plan-Apochromat 10x/0.3 M27 (Zeiss), sciatic nerves were imaged, before and after injury, with a frame acquisition rate consistent across comparable data sets. Injury was performed by the compression of the sciatic nerve, in the distal part, for 30 s with a flat handle micro jewellers forceps. One millimolar H_2_O_2_ was added directly above the sciatic nerve by drops (total volume 20 μl) at the end of the recordings as a positive control. The mitochondria-targeted antioxidant MitoTEMPO (SML0737, Sigma-Aldrich) or the NOX inhibitor VAS2870 (492,000 Calbiochem) were applied (1 mM) in the same way before injury. Imaging was completed within 20 min of anaesthesia. A minimum of three sciatic nerves were imaged per condition. Mean fluorescence was measured by averaging the intensity across the visible sciatic nerve area.

### Primary cell cultures and treatments

Primary cultures of SCs and of spinal cord motor neurons (SCMNs), and the relative co-cultures, were prepared as previously described [20, 43]. Primary SCs were exposed to 50 µM H_2_O_2_ in Krebs Ringer Buffer (KRH: HEPES-Na 25 mM at pH 7.4, NaCl 124 mM, KCl 5 mM, MgSO_4_ 1.25 mM, CaCl_2_ 1.25 mM, KH_2_PO_4_ 1.25 mM, glucose 8 mM) for different amounts of time at 37 °C, and then processed for immunofluorescence or ELISA.

Co-cultures between SCs and SCMNs were kept for 2–3 days in SCMNs medium before treatment, then exposed for different times to α-LTx (0.1 nM) in E4 medium (120 mM NaCl, 3 mM KCl, 2 mM MgSO_4_, 2 mM CaCl_2_, 10 mM glucose, and 10 mM HEPES-Na, pH 7.4) at 37 °C, and processed for immunofluorescence.

### Immunofluorescence

Samples were exposed to H_2_O_2_ with or without the Transcriptional enhancer factor domain (TEAD) inhibitor VT107 (HY-134957 MedChemexpress, 10 μM final concentration). VT107 is a potent inhibitor of TEAD4 palmitoylation [44]. All TEAD homologues require auto-palmitoylation on the sulfydryl of a conserved cysteine to become functional. Samples were then fixed for 15 min in 4% PFA in PBS, quenched (0.24% NH_4_Cl in PBS), permeabilized with 0.3% Triton X100 in PBS for 5 min at RT, and saturated with 3% goat serum in PBS for 1 h. After incubation with primary antibodies diluted in 3% goat serum in PBS overnight at 4˚C, samples were washed and then incubated with the corresponding AlexaFluor-conjugated secondary antibodies for 1 h at RT. Coverslips were mounted in Mowiol and examined by confocal (Leica SP5) microscopy. Fluorescence intensity was quantified using Fiji Software. Images were acquired using non-saturating settings, and the same imaging parameters were used for all samples.

### ELISA

Ctgf was quantified in the supernatant of primary SCs cultured in 24-well plates and exposed for different time periods to 50 µM H_2_O_2_. Briefly, the supernatant was collected and centrifuged at 12,000 × g at 4 °C to eliminate cell debris. Aliquots were immediately assayed according to manufacturer’s instructions (Peprotech 900-M317). Ctgf standards were used in the range of 4 to 4000 pg/ml.

#### In vitro scratch wounding assay

SCs were seeded onto 35 mm Glass Bottom Dishes (Mattek) on a coating of laminin (3 μg/ml) w/wo 100 ng/ml of recombinant human Ctgf (rCtgf, 120–19 Peprotech). Once fully confluent, the monolayers were scratched using a sterile 200 μl pipette tip following the protocol described in [45]. Monolayers were then gently washed twice with DMEM to remove cell debris, and incubated at 37 °C (5% CO_2_ air atmosphere). Images were acquired 0, 8 and 24 h later. The wound scratch area was measured using ImageJ.

### Statistical analysis

Sample size was determined based on data collected in our previously published studies. We used at least N = 4 mice/group for electrophysiological analysis. For imaging and cell cultures studies, at least three independent replicates were performed. Mice were randomly allocated to the different treatment groups by the investigator. We ensured blinded conduct of experiments. For imaging analysis, the quantitation was conducted by an observer who was blind to the experimental groups. No samples or animals were excluded from the analysis. Data displayed in histograms are expressed as means ± SD. GraphPad Prism software was used for all statistical analyses except for RNAseq data, where the glmQLFTest function, which applies a quasi-likelihood F-test, was employed. Statistical significance was evaluated using unpaired Student’s *t*-test, or paired Student’s *t*-test for live-imaging experiments. Data were considered statistically different when *p < 0.05, **p < 0.01, ***p < 0.001.

## Results

### Nerve injury induces upregulation of ECM components at the regenerating NMJ

To profile the NMJ transcriptome during α-LTx-induced degeneration and the subsequent recovery, we injected the hind limb of mice with the toxin, and collected soleus muscles 4, 16, 72 and 168 h post-treatment. Contralateral, control muscles received saline only. These time points encapsulate the period of MAT degeneration and regeneration in soleus muscles as determined by immunostaining of the pre-synaptic marker Synaptosomal-Associated Protein, 25 kDa (SNAP25) and neurofilaments (NF) (Additional file 1: Fig. S1): 4 h (MAT degeneration with active phagocytosis by PSCs), 16 h (clearance of neuronal debris almost complete), 72 h (ongoing MAT re-growth), and 168 h (morphological recovery) [20]. To visualise endplates, fluorescent α-BTx was bilaterally injected into soleus muscles prior to their dissection and rapid freezing. Sections were then cut, and one hundred NMJs/sample were collected by LCM, and processed for sequencing (Fig. 1A).

To understand the global changes occurring during MAT degeneration and regeneration, transcriptional states were first investigated by multi-dimensional scaling analysis (MDS), which summarises the similarity between each time point, based on the levels of all expressed genes. Notably, the degeneration and regeneration cycle is captured by the dynamic changes in NMJ transcriptomes, resulting in a ring-shaped configuration, where chronological stages follow a circular trajectory (Fig. 1B). Whilst the regeneration profile at 72 h is clearly distinct from that of a mature resting NMJ, at later stage of regeneration the transcriptome profile is approaching that of the pre-injury control state. This trend is consistent with the time-course of MAT degeneration and regeneration (Additional file 1: Fig. S1), and is further supported by the number of differentially expressed genes (DEGs) identified at the different time points (Fig. 1C, and Additional file 2). Differential expression analysis shows that the largest transcriptional variations occur between the 16 and 72 h time points, when the NMJ undergoes major morphological transformation (*i.e.*, phagocytosis of terminal debris by PSCs, axonal elongation, re-establishment of synaptic contacts). At the last time point analysed, the number of DEGs decreases, reflecting the anatomical recovery of the MAT.

Gene ontology and REACTOME pathway enrichment analyses reveal that genes largely related to metabolism and muscle development are downregulated, while genes involved in ECM homeostasis display a global increment (Additional file 1: Fig. S2 and Additional file 3). Among the latter, both regulatory members of the ECM (e.g., the matricellular proteins tenascin C, thrombospondins, Ctgf, osteopontin, periostin, Wisp 1 and Wisp 2) and structural components (*e.g.*, different collagen isoforms) show an overall increase during regeneration. At variance from signalling members of the ECM, whose levels approach the baseline at the latest time point (168 h), the increment of collagens is kept high for a longer time (Additional file 1: Fig. S3A, B).

Thus, a striking remodelling of the ECM occurs during NMJ degeneration and recovery to support PSC activation, axonal re-growth and, ultimately, rescue of neurotransmission.

### Hydrogen peroxide signaling at the regenerating NMJ

We previously reported that H_2_O_2_ is produced by mitochondria of stressed neurons and is a major SC activator [20, 22, 23]. We therefore wanted to determine whether this important *alarm* molecule was driving some of the transcriptomic changes we observed at the regenerating NMJ.

**Fig. 2:**
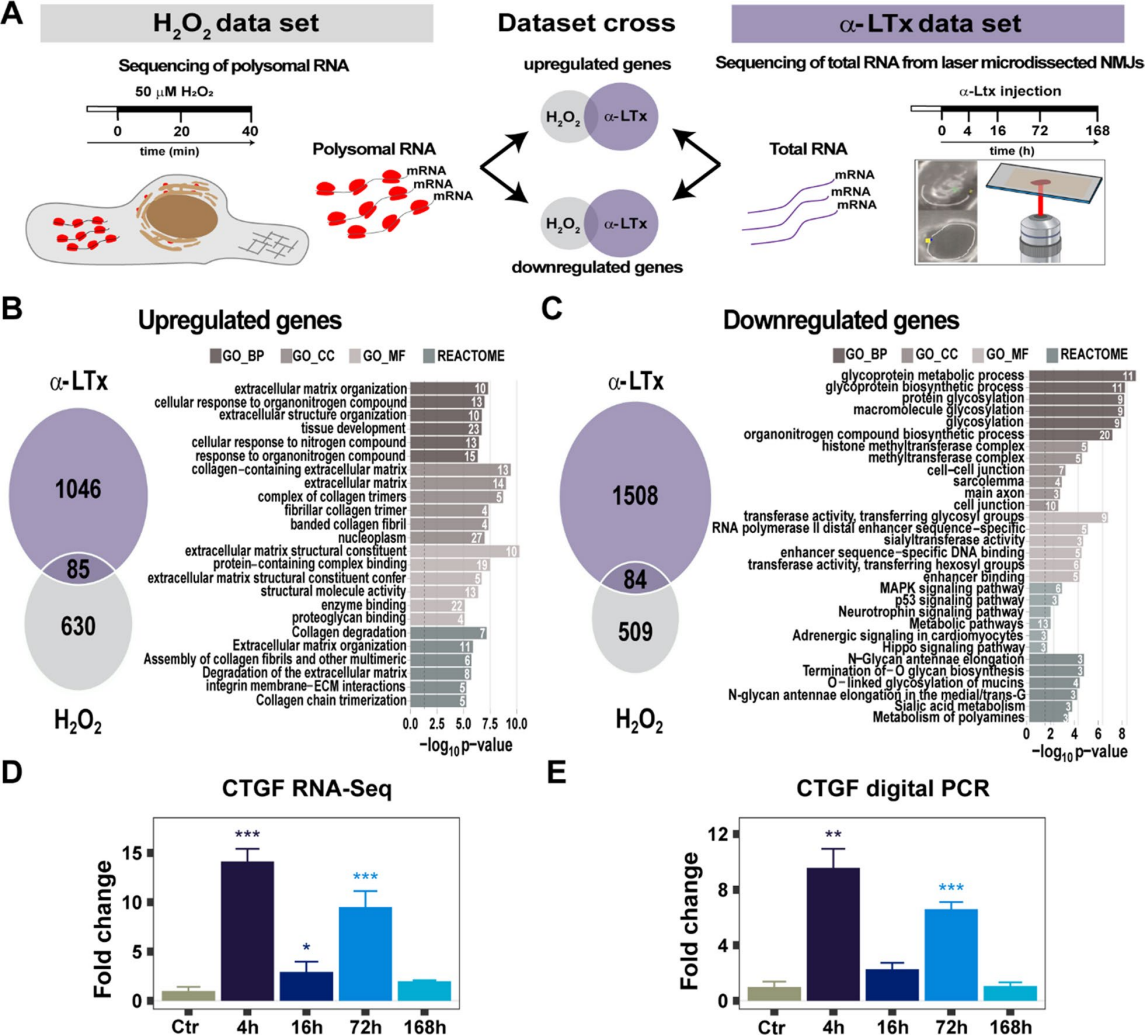
ECM components, including *Ctgf*, are enriched at the regenerating NMJ. **A** Schematic overview of the integration approach used to identify genes critical to regeneration. The H_2_O_2_ data set (left) was obtained by polysome sequencing of SCs exposed to H_2_O_2_ for 20 and 40 min [10] and the α-LTx time-course (right) as outlined in Fig. 1. **B**, **C** Venn diagrams showing the total number of DEGs up- (**B**) or down- (**C**) regulated in the toxin and in the H_2_O_2_ data sets, the DEGs in common (169, 85 up vs 84 down), and the relative gene ontology and REACTOME pathway enrichment analysis. The number of genes associated with each category is displayed on the right of the bars (GO_BP: biological process, GO_CC: cellular component, GO_MF: molecular function). **D**, **E** RNA seq (**D**) and digital PCR (**E**) quantification of *Ctgf* mRNA levels during the time-course of α-LTx treatment, normalized to control. The reported significant differences were determined by differential expression analysis. N = 5, *p = 0.010, **p = 0.002, ***p < 0.001

We thus cross compared the α-LTx-induced NMJ DEGs with our previously published data set of actively translated RNAs associated with polysomes purified from H_2_O_2_-treated primary SCs [23] (Fig. 2A). Common genes were defined by the intersection of genes up- or down-regulated in at least one time point of both α-LTx and H_2_O_2_ treatments. A total of 169 common DEGs (85 up and 84 down, Additional file 4) were identified, and the relative Gene Ontology enrichments are herein reported (Fig. 2B, C and Additional file 5). The 85 common up-regulated genes are enriched in structural and signalling ECM components (Fig. 2B, and Additional file 6). Among the top common up-regulated genes, we focused on the transcript encoding the matricellular protein Ctgf, whose involvement in peripheral nerve regeneration in mice had not been previously addressed. Noticeably, at the intoxicated NMJ, up-regulation of *Ctgf* mRNA follows a biphasic trend, with an early peak, during the acute phase of degeneration (4 h), and a second increase 72 h post-intoxication, during regeneration (Fig. 2D). This trend was confirmed by digital PCR (Fig. 2E). Remarkably, *Ctgf* up-regulation at the NMJ matches the previously reported trend of *Ctgf* mRNA during nerve regeneration in *bridge* SCs [46] (Additional file 1: Fig. S3C). Consistent with a possible role of Ctgf in regeneration at the neuromuscular synapse, most Ctgf receptors (α_*V*_β_3_, α_6_β_1_, α_5_β_1_, p75NTR, LRP-1, LRP-6) are expressed in our soleus NMJ data set.

These results suggest that H_2_O_2_ triggers ECM remodeling at the regenerating NMJ, and that temporal waves of *Ctgf* up-regulation may contribute to this process.

### Ctgf localizes to the murine NMJ and promotes its functional recovery upon injury

To assess the presence of Ctgf at the NMJ, we performed immunofluorescence analysis of the *levator auris longus* (LAL), a thin muscle suitable for whole mount imaging [47]. In non-injured NMJs, we determined that Ctgf localizes to PSCs and in the extracellular space (Additional file 1: Fig. S4A upper panels), as confirmed by orthogonal projections and 3D reconstructions (Additional file 1: Fig. S4B, C and Additional file 7: Movie 1).

MAT degeneration and regeneration induced by α-LTx results in the progressive loss and reappearance of NF (Additional file 1: Figs. S1, S4A). During this process, we identified that Ctgf is redistributed at the synapse, becoming more extracellular, as shown by immunofluorescence (Additional file 1: Fig. S4A lower panels), and 3D reconstructions of α-LTx-treated NMJs (Additional file 8: Movie 2).

The increase in *Ctgf* transcript (Fig. 2D, E) is likely to be initiated by *alarm* signals (including H_2_O_2_) released by degenerating motor axons that activate SCs [20, 22, 23, 48]. In support of this possibility, we found that α-LTx exposure increases Ctgf availability in SCs co-cultured with spinal cord motor neurons (SCMNs) (Additional file 1: Fig. S5A, B), and that H_2_O_2_ treatment similarly rapidly increases Ctgf levels and release (Additional file 1: Fig. S5C–E).

**Fig. 3:**
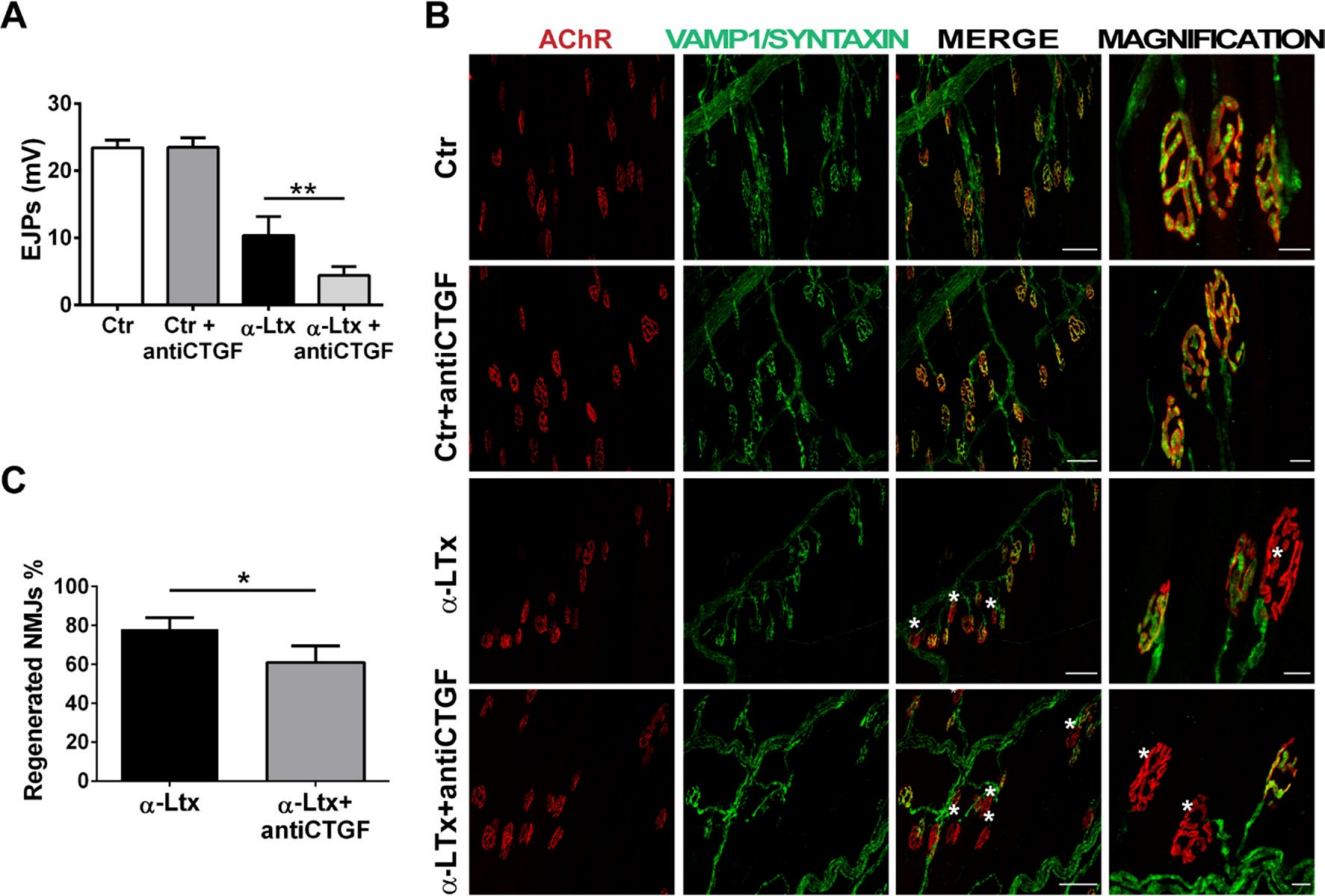
Ctgf neutralization delays functional and morphological recovery of the NMJ. **A** Evoked junctional potentials (EJPs) recorded at soleus NMJs treated with α-LTx for 96 h, in the absence or presence of a neutralizing antibody against Ctgf. N = 5, 15 fibers analyzed. **p = 0.00**. B** Muscles in **A** were processed for indirect immunofluorescence using fluorescent α-BTx to stain post-synaptic AChRs (*red*), and combined anti-syntaxin 1A/1B and anti-VAMP1 antibodies to identify the pre-synaptic compartment (*green*). Asterisks identify degenerated NMJs. Scale bars: 50 µm (10 µm in magnification). **C** Quantification of regenerated NMJs. N = 5, one hundred NMJs analyzed/muscle. *p = 0.048

To assess the contribution of Ctgf to MAT regeneration, we intraperitoneally injected a Ctgf-neutralizing IgG monoclonal antibody before the local injection of α-LTx in the hind limb of mice. Neurotransmission was evaluated by recording the evoked junctional potentials (EJPs) of soleus muscle fibres 96 h later, when ≈50% of neurotransmission is rescued at intoxicated NMJs. We discovered that functional recovery of the NMJ is significantly delayed upon Ctgf neutralization (Fig. 3A). This result is corroborated by immunostaining of the pre-synaptic markers syntaxin and VAMP1, which showed that Ctgf neutralization also impaired anatomical rescue, with fewer NMJs displaying re-innervation (Fig. 3B, C). It is important to note that, as expected, there is only a partial impairment in regeneration upon Ctgf neutralization, as additional factors contribute to the process [1–4, 6, 7, 9, 10].

### Local hydrogen peroxide production upon nerve injury drives Ctgf production

**Fig. 4:**
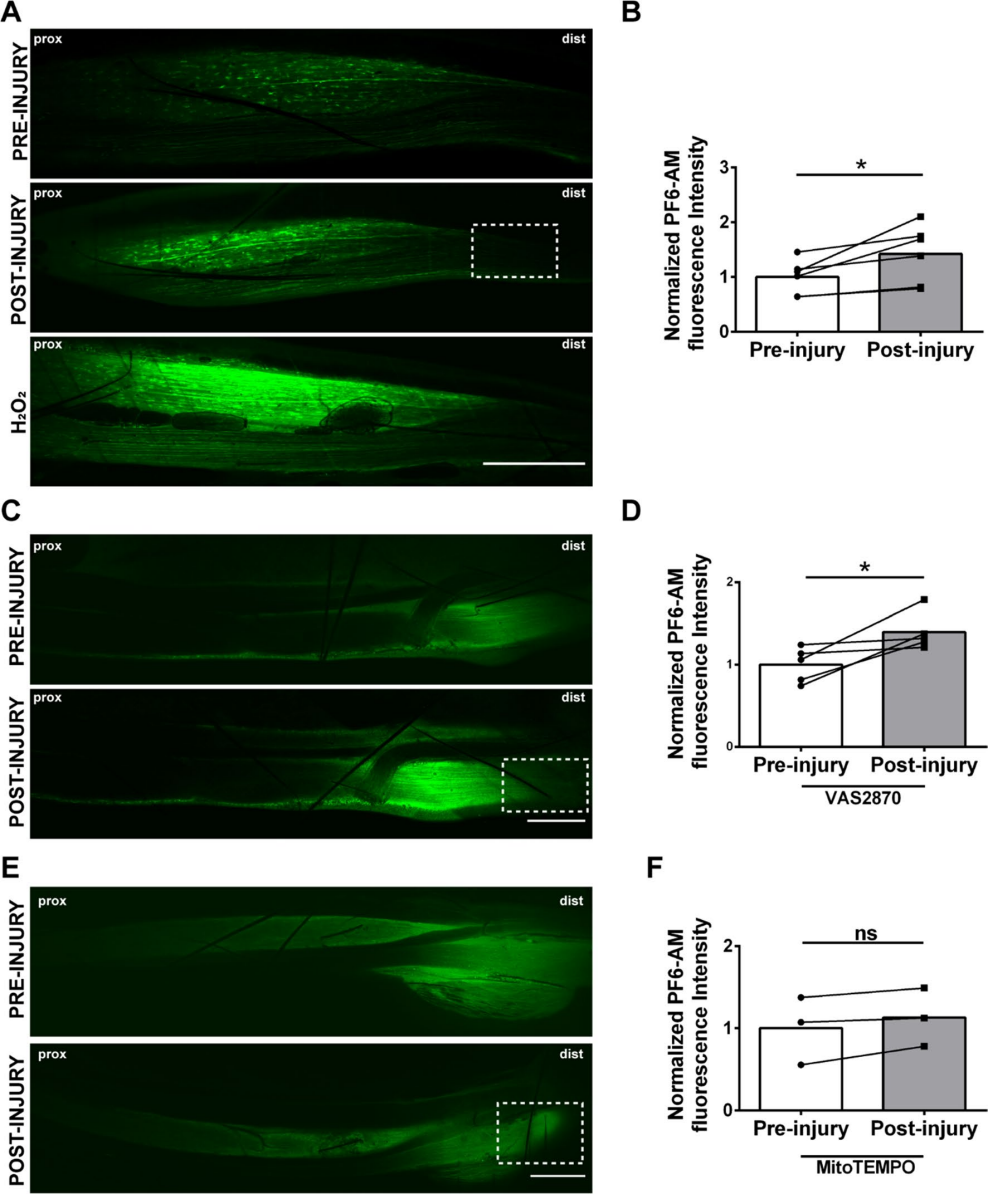
H_2_O_2_ is rapidly produced upon compression of the sciatic nerve and originates from mitochondria. **A, B** Imaging of H_2_O_2_ production in the sciatic nerve of a live, anaesthetized mouse before (**A**, upper panel) and after (**A**, middle panel) sciatic nerve crush, and quantification of PF6-AM signal (**B**, *p = 0.029). The dotted squares indicate the lesion site. Lower panel: PF6-AM fluorescence in response to 1 mM H_2_O_2_ (positive control). Scale bar: 500 µm. **C**, **D** H_2_O_2_ increase following sciatic nerve crush is unaffected by the NOX inhibitor VAS2870. Scale bar: 500 µm. Quantification of PF6-AM signal is shown in **D**. N = 3, *p = 0.045. **E**, **F** H_2_O_2_ increase upon sciatic nerve crush is abolished by MitoTEMPO incubation. Scale bar: 500 µm. Quantification of PF6-AM signal in shown in **F**. N = 3, *p* = 0.123, ns = not significant

To extend our findings at the NMJ to additional models of nerve damage and evaluate the possibility that the local production of H_2_O_2_ by neurons is a general injury response, we assessed H_2_O_2_ levels in the sciatic nerve of live, anaesthetized mice before and after nerve compression by injecting the H_2_O_2_-specific fluorescent probe PF6-AM [42] beneath the sciatic nerve epineurium. Strikingly, nerve crush induces H_2_O_2_ generation close to the injury site within two minutes of damage (Fig. 4A, B). At the end of each experiment, 1 mM H_2_O_2_ was applied as a positive control (A, bottom panel).

To shed light on the origin of H_2_O_2_, we bathed the sciatic nerve prior to crush in either the NADPH oxidase (NOX) inhibitor VAS2870 or the mitochondria-targeted antioxidant MitoTEMPO. Inhibition of NOX activity had no impact on the injury-induced increase in H_2_O_2_ (Fig. 4C, D), while MitoTEMPO treatment restricted the production of this *alarm* signal (Fig. 4E, F), indicating that the upsurge in H_2_O_2_ signaling in the damaged sciatic nerve is derived from mitochondria, a finding in agreement with our previous observations at the intoxicated MAT [20].

**Fig. 5:**
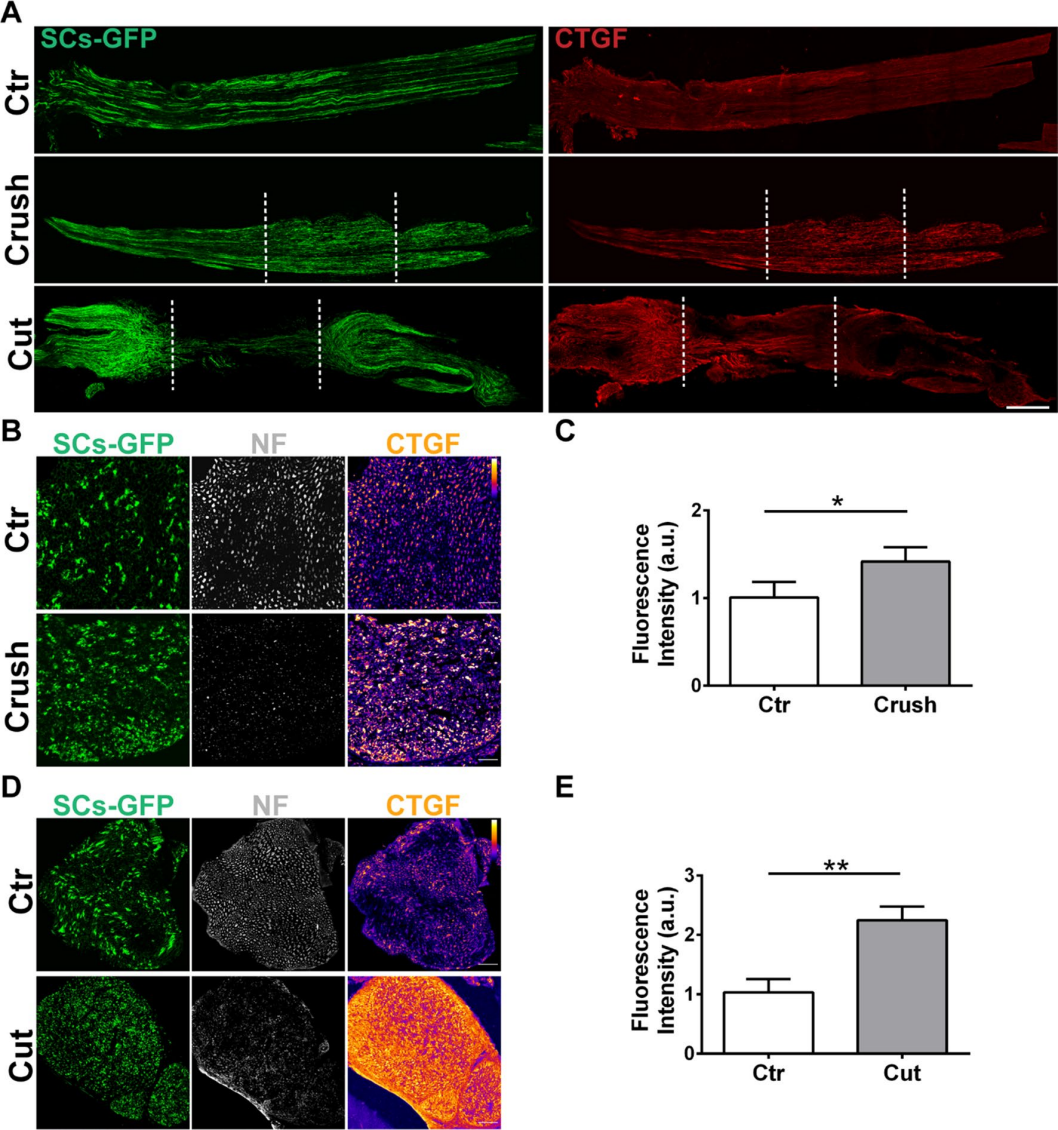
Sciatic nerve injury drives increased Ctgf levels. **A** Longitudinal views of whole mount preparations of sciatic nerves from control mice (Ctr, upper panels), four days after nerve compression (crush, middle panels), and six days after nerve transection (cut, lower panels). Dotted lines indicate the injury site. Ctgf is in *red* and SCs are GFP-positive (*green*). Scale bar: 100 µm. **B**, **D** Cross-sections of sciatic nerves in control conditions (Ctr, upper panels), four days after nerve compression (**B**), and six days after nerve transection (**D**). SCs are GFP-positive (*green*), NF in *white*, Ctgf is in pseudocolors. A pseudocolor scale is added in the corresponding figures, where *white* corresponds to the highest intensity, and *black* to the lowest. Scale bars: 50 µm. **C**, **E** Quantification of Ctgf signal in cross sections. N = 3, *p = 0.041 (**C**), **p = 0.003 (**E**)

Since toxin-induced nerve injury results in increased Ctgf availability at the regenerating NMJ (Fig. 2D, E and Additional file 1: Fig. S4A), we assessed whether a similar upregulation in Ctgf occurs upon sciatic nerve injury. Whole mount immunofluorescence analysis showed strong increases in Ctgf following either a compression or a transection of the sciatic nerve (Fig. 5A, longitudinal view). These findings were replicated in analyses of nerve cross-sections collected over the lesion site, both in response to compression (Fig. 5B, C) and to transection (Fig. 5D, E). Confirming successful lesions, we observed NF loss due to motor axon degeneration, and a global Ctgf increase in myelinating SCs, as well as in other cell types within the nerve (Fig. 5B–E).

**Fig. 6:**
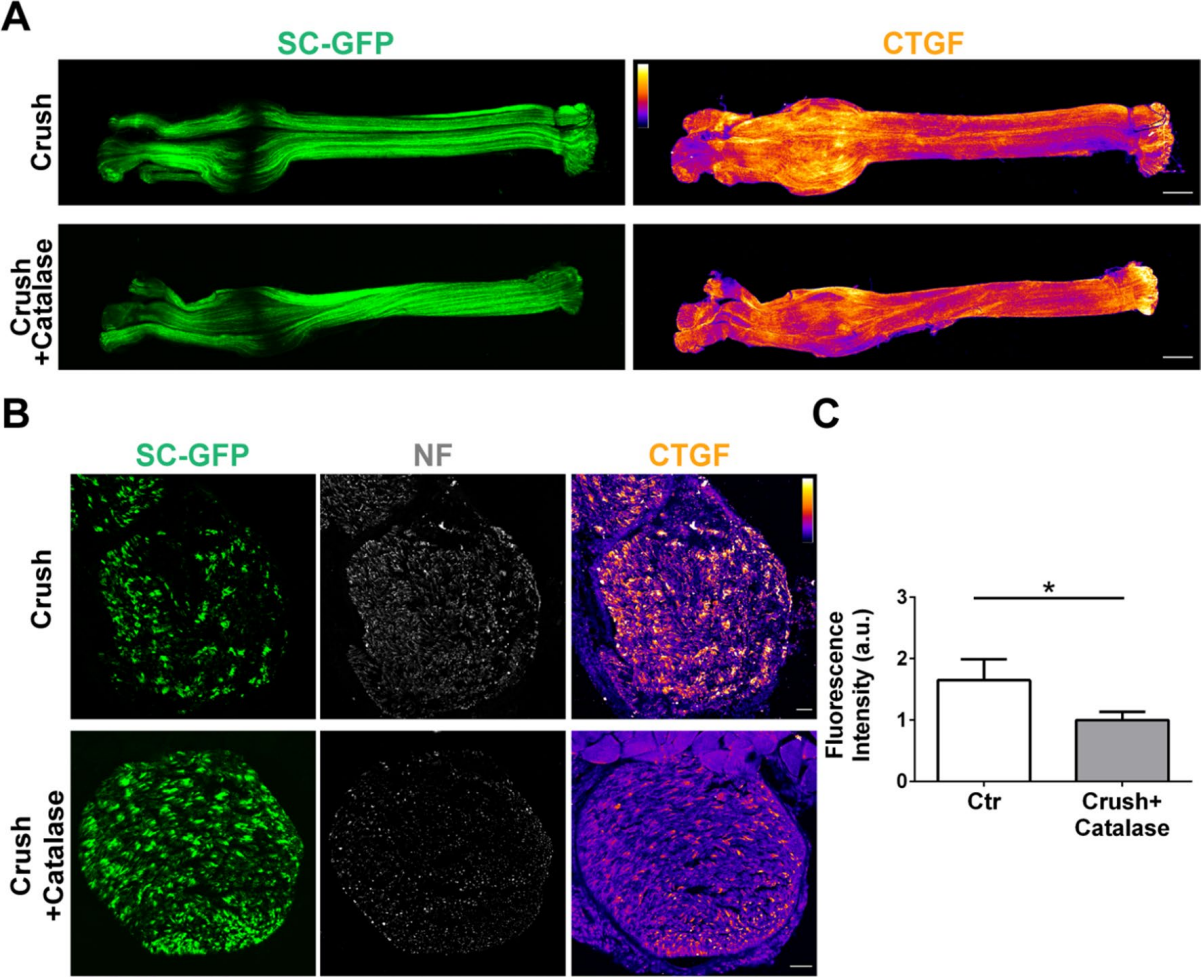
Injury-induced Ctgf production is driven by H_2_O_2_. **A** Longitudinal view of whole mount preparations of sciatic nerves four days after nerve crush in the absence (upper panels) or presence (lower panels) of catalase delivered intra-sciatically. Ctgf is in pseudocolors  and SCs are GFP-positive (*green*). Scale bars: 100 µm. **B** Cross-sections of sciatic nerves four days after nerve compression in the absence (upper panels) or presence (lower panels) of catalase delivered via intra-nerve injection. SCs are GFP-positive, NF are in *white* and Ctgf is in pseudocolors. A pseudocolor scale is added in the corresponding figures, where *white* corresponds to the highest intensity, and *black* to the lowest. Scale bars: 100 µm. Quantification of Ctgf signal in cross-sections is shown in **C**. N = 4, *p = 0.012

To determine the role of H_2_O_2_ in the local upregulation of Ctgf in response to nerve lesion, we pre-treated nerves by injection with catalase, which restricts H_2_O_2_ availability by converting it to H_2_O and O_2_. Catalase treatment dampened the increase in Ctgf levels (Fig. 6A, longitudinal view, and 6B-C, cross sections and relative quantification), indicating that H_2_O_2_ is an important mediator of the Ctgf production.

### Ctgf promotes neurotransmission recovery and polarized axonal elongation in the injured sciatic nerve

**Fig. 7:**
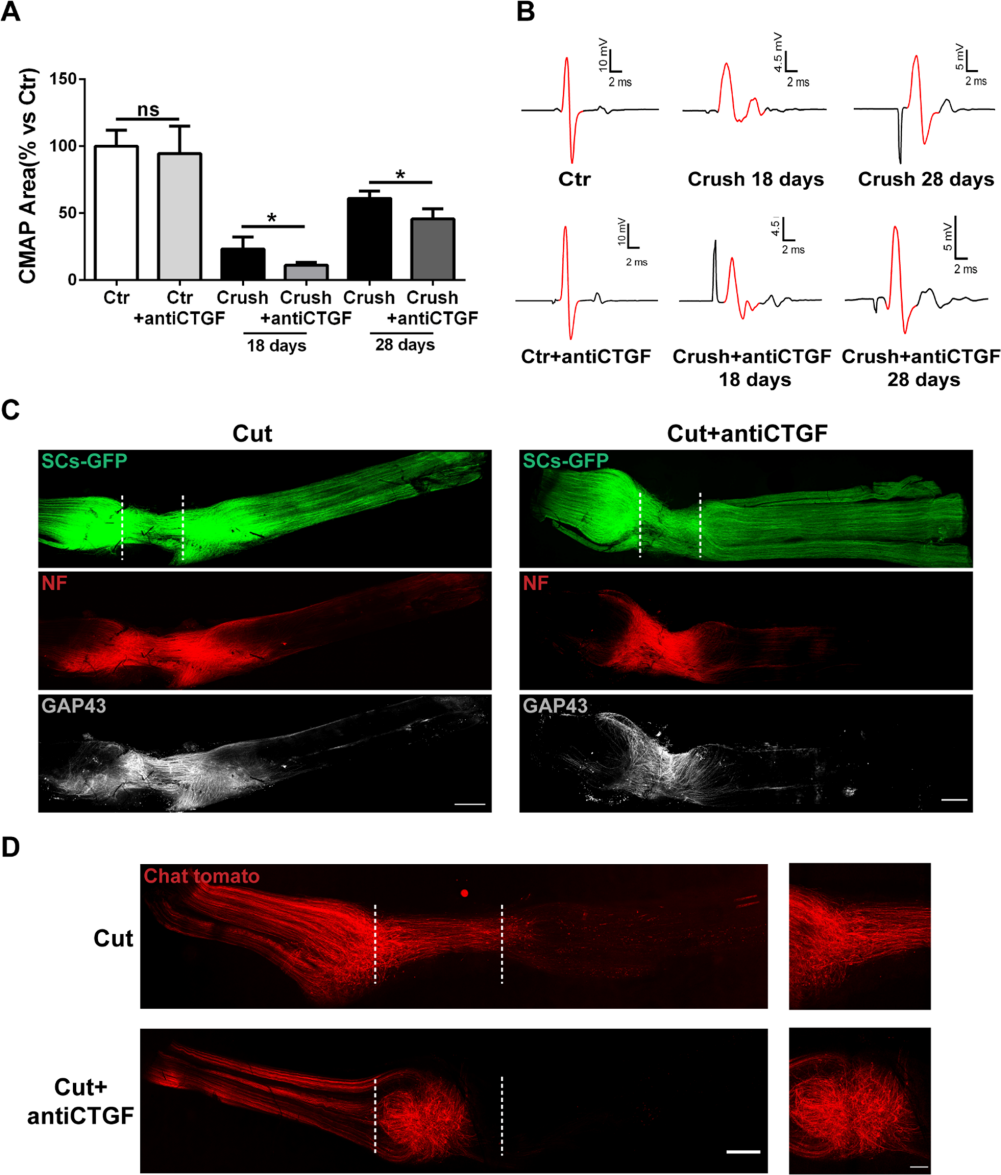
Ctgf neutralization delays neurotransmission recovery upon sciatic nerve injury. **A** CMAP amplitude recordings in control gastrocnemius muscles, as well as 18 and 28 days after compression of the sciatic nerve, in the absence or presence of a neutralizing antibody against Ctgf. N = 4, 20 fibers analyzed. *p = 0.038 (18 days), *p = 0.042 (28 days). **B** Representative CMAP traces of control and crushed muscles 18 and 28 days after injury, w/wo anti-Ctgf intraperitoneal administration, showing polyphasic features in muscles treated with the neutralizing antibody. The red traces show the portions used to calculate the CMAP area, measured from the initial to the terminal deflection back to baseline. **C, D** Axonal re-growth, monitored by NF (*red*) and GAP43 (*white*) immunoreactivity (**C**), and by Tomato expression (*red*) in Chat-positive motor axons (**D**), 12 days (**C**) and 10 days (**D**) after nerve transection. SCs are in *green* (**C**). Dotted lines indicate the injury site. Nerve re-growth is reduced and less organized when Ctgf is neutralized. Scale bars: 500 µm (200 µm in the magnification)

Ctgf plays a role in functional and anatomical recovery at the intoxicated NMJ (Fig. 3); we thus performed antibody neutralization experiments to determine whether the increased Ctgf availability upon sciatic nerve injury aids regeneration. To do so, we intraperitoneally injected anti-Ctgf neutralizing antibody once per week after nerve crush, and assessed functional recovery at 18 and 28 days. Reducing Ctgf availability delayed neurotransmission rescue of crushed sciatic nerves at both time points, as shown by electromyographic recordings of the compound muscle action potentials (CMAP) in gastrocnemius muscles (Fig. 7A). In contrast with untreated mice, CMAP traces from injured sciatic nerves treated with the Ctgf-neutralizing antibody maintain a disrupted, polyphasic shape 28 days post-nerve damage, further supportive of delayed recovery (Fig. 7B).

We also assessed regeneration with and without Ctgf neutralisation after nerve transection. In line with findings post-crush, Ctgf neutralisation caused reduced and misdirected motor axon elongation after cut, as shown by NF and GAP-43 (growth-associated protein) distribution in whole mount nerve preparations (Fig. 7C), and by Tomato-expressing ChAT-positive axons that underwent the same experimental protocol (Fig. 7D).

### Ctgf aids polarized axonal re-growth by guiding SC migration

Neurotransmission blockade by α-LTx leads to a change in the shape of PSCs that extend processes or sprouts emerging from the paralyzed endplates. This response is particularly evident in slow-twitch muscles like the soleus (Additional file 1: Fig. S6A, upper panels), and assists and drives the sprouting of neurons [16, 49]. Sprouts represent a plastic, compensatory response of PSCs and motor axons to neurotransmission impairment, and mark the initiation of motor axon regeneration. Strikingly, we observed that PSC sprouts are Ctgf-positive (Additional file 1: Fig. S6A, upper panels, and Additional file 8: Movie 2). 3D reconstructions in Additional file 1: Fig. S6B allows a better visualization of Ctgf-positive sprouts in PSCs. These observations suggest that this protein may exert a pro-regenerative activity by sustaining/guiding axonal growth.

**Fig. 8:**
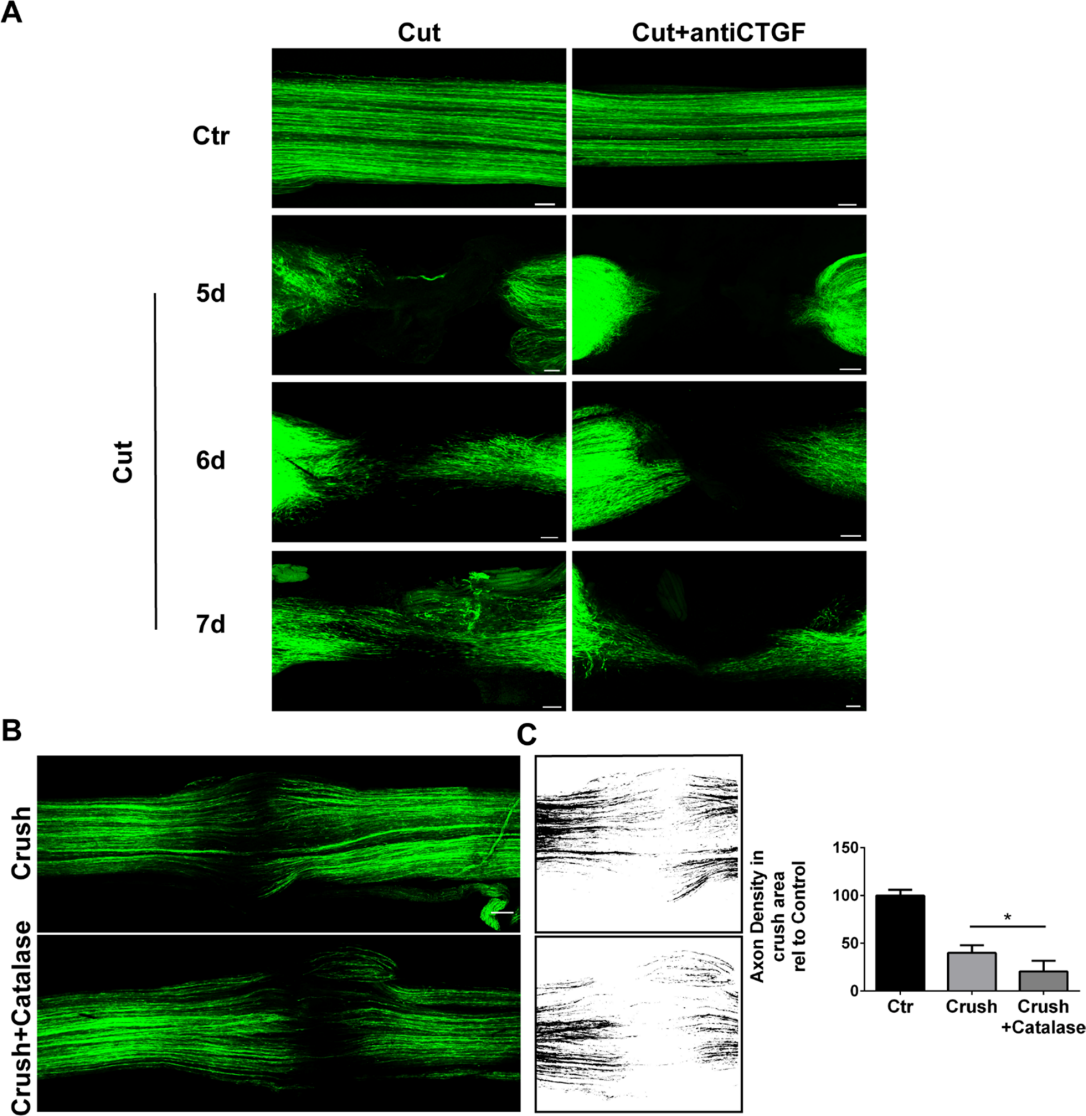
Hydrogen peroxide-induced Ctgf facilitates the effective migration of SCs to sustain axonal re-growth. **A** Magnification showing SC (GFP-positive, *green*) migration across the bridge upon sciatic nerve cut (five to seven days) in the absence (left panels) or presence (right panels) of an anti-Ctgf neutralizing antibody. Scale bars: 100 µm**. B**, **C** Intra-sciatic delivery of catalase slows down SC migration across the bridge in regenerating nerves (four days after crush). Scale bar: 100 µm. Axon density in the bridge was quantified by converting fluorescent images to black and white and measuring pixel density in the selected crush area (**C**). N = 3, *p = 0.031

To test this hypothesis, we paralyzed the NMJ either by denervation (Additional file 1: Fig. S6A, middle panels), or by treatment with botulinum neurotoxin A (BoNT/A) (Additional file 1: Fig. S6A, lower panels), and monitored the presence of Ctgf in sprouts. In all the three conditions tested (reversible degeneration by α-LTx, sciatic nerve transection, and functional denervation by BoNT/A), PSC processes express Ctgf, suggesting that the molecule may indeed support PSC sprouting and, in turn, axonal elongation. Accordingly, Ctgf neutralization reduces the sprouts emerging from the MAT paralyzed by BoNT/A (Additional file 1: Fig. S6C, D). Mirroring this and providing further evidence of the ability of Ctgf to support SC migration, we observed faster wound healing upon scratching by SCs plated on a coating of recombinant Ctgf plus laminin, compared to SCs grown on laminin alone (Additional file 1: Fig. S6E, F). To extend these findings in vivo, we performed nerve transection experiments and showed that Ctgf neutralisation delays SC migration along the bridge, which is the new tissue that forms after cutting to reconnect the two axonal stumps (Fig. 8A). Moreover, and in line with the H_2_O_2_-dependent expression of Ctgf, H_2_O_2_ neutralization by treatment with catalase slows down SC migration across the bridge in regenerating nerves (Fig. 8B, C).

Overall, these findings support the hypothesis that H_2_O_2_ and its downstream product Ctgf are pro-regenerative factors that guide axonal growth along the trajectories provided by Ctgf-expressing SCs.

### H_2_O_2_ signals through the YAP pathway to drive *Ctgf* transcription

**Fig. 9:**
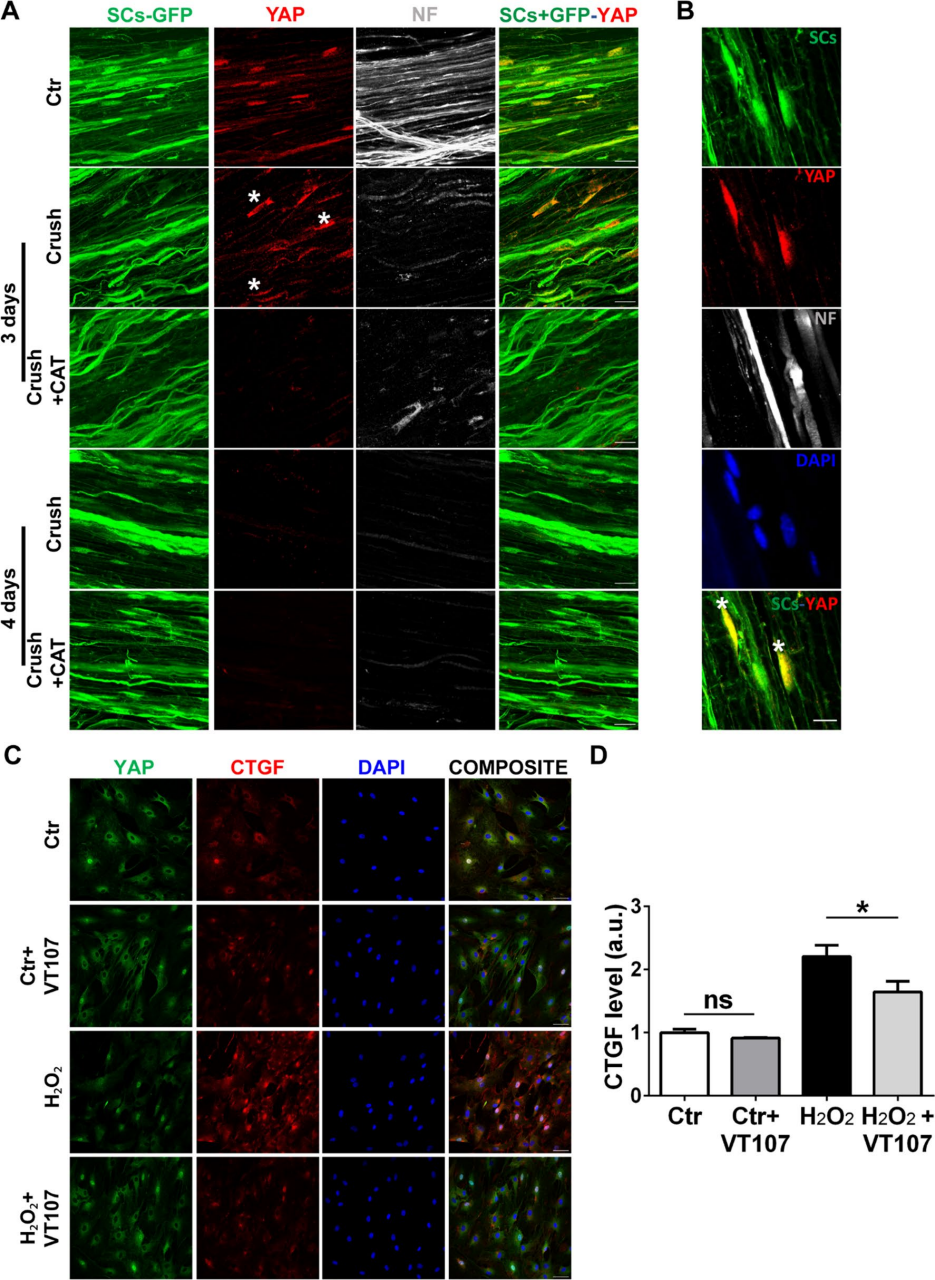
Ctgf production upon nerve injury is H_2_O_2_- and YAP-dependent. **A** YAP (*red*) expression and localization in SCs (expressing GFP under the *plp*-promoter, *green*) in control nerves and 3/4 days post-crush, in the absence or presence of catalase delivered into the sciatic nerve. NF is in *white* and nuclei are stained with DAPI *(blue).* Asterisks mark YAP-positive SCs. Scale bars: 20 µm. A magnification of YAP localization in control nerves in shown in panel **B**. Scale bar: 10 µm. **C**, **D** Representative immunostaining of YAP and Ctgf in primary SCs exposed to 50 µM H_2_O_2_ w/wo the pan-TEAD autopalmitoylation inhibitor VT107 (**C**). YAP is in *green*, Ctgf in *red* and nuclei are in *blue*. Scale bars: 50 µm. Quantification of Ctgf levels by ELISA is shown in **D**. N = 3, *p = 0.0166, ns = not significant

Several signaling pathways converge on *Ctgf* transcription, including the transcription factors TEADs (Hippo signaling effectors) with their co-activators and Hippo transducers YAP/TAZ [50]. Given the emerging importance of mechanosignaling for SC biology during development and in nerve regeneration, and in light of the fact that TEAD4 is upregulated in the NMJ transcriptome (Additional file 1: Fig. S3D), we looked at YAP expression and localization in response to H_2_O_2_ both in vivo and in vitro (Fig. 9). In the adult sciatic nerve, YAP expression in SCs is innervation-dependent (Fig. 9A upper panels and panel B); indeed, YAP disappears from denervated SCs, but reappears in SC nuclei during axonal regeneration [51]. To determine whether H_2_O_2_ generated at the lesion site impacts the YAP signal in SC nuclei, we performed pre-crush intra-sciatic nerve injections of catalase and assessed YAP localization three and four days post-injury. YAP is still present in SCs nuclei three days after crush, when neurons start degenerating, and disappears the day after. Catalase treatment caused YAP to disappear from SCs already on day three after damage (Fig. 9A), suggesting that H_2_O_2_ sustains YAP transcriptional activity, which in turn drives *Ctgf* expression. Finally, we cultured SCs and exposed them to H_2_O_2_ in the presence of VT107, a pan-TEAD autopalmitoylation inhibitor. VT107 treatment dampened the H_2_O_2_-mediated increase in Ctgf levels (Fig. 9C, D), suggesting that TEAD transcription factors are required to fully activate *Ctgf* expression.

Together, these findings indicate that production of the pro-regenerative factor Ctgf in SCs is both H_2_O_2_- and YAP-dependent.

## Discussion

**Fig. 10:**
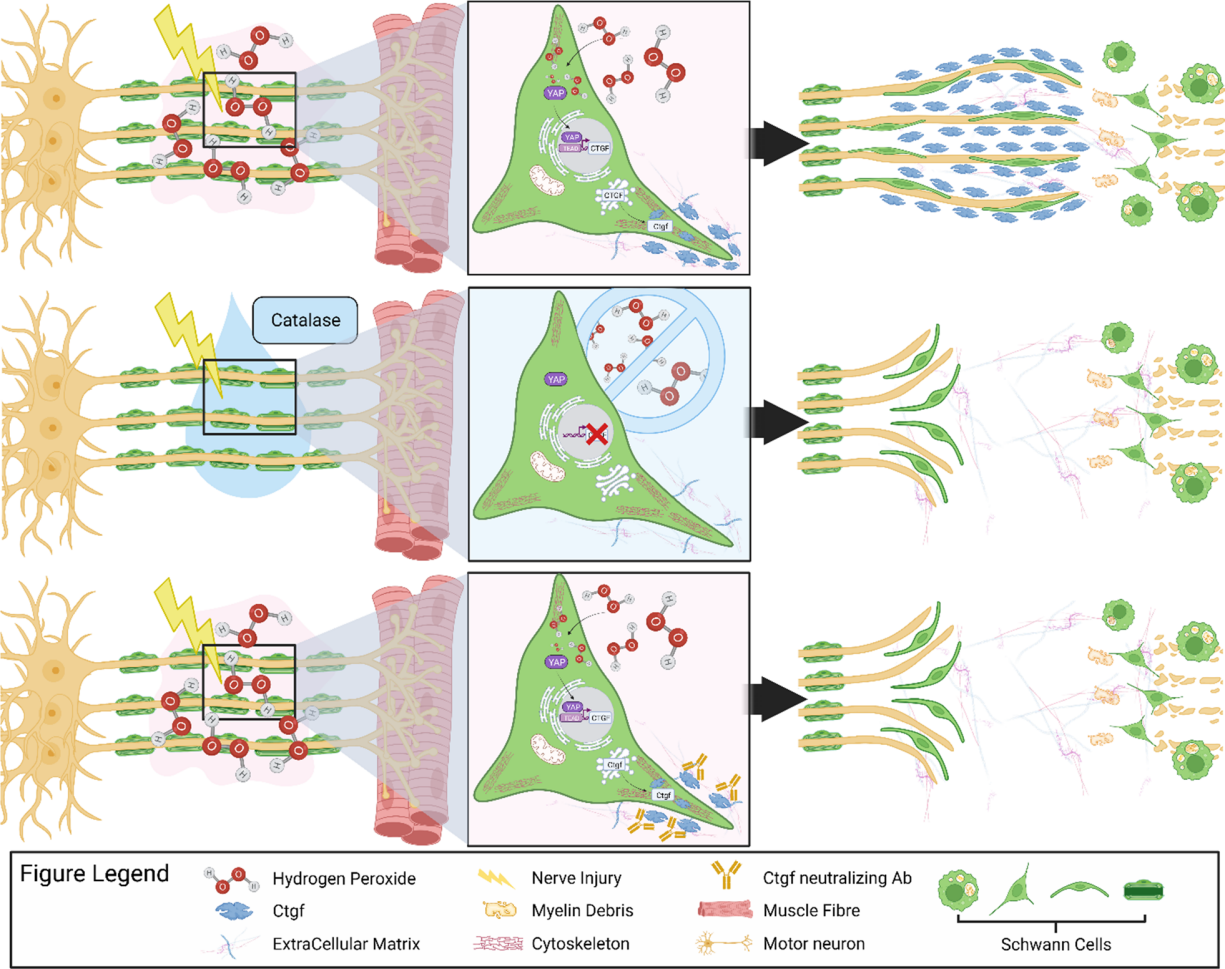
H_2_O_2_ signaling and Ctgf production are critical for efficient nerve repair. Schematic representation of the local H_2_O_2_ signaling triggered by acute peripheral nerve injury and its role in nerve recovery. *Upper panels*: acute damage to the sciatic nerve triggers a rapid, local production of H_2_O_2_ that, in turn, stimulates the YAP-dependent expression of Ctgf by SCs, promoting efficient and polarized nerve regeneration. *Middle panels*: H_2_O_2_ neutralization by catalase inhibits Ctgf expression, causing a misdirected migration of SCs and disorganised axonal re-growth. *Lower panels*: The same misdirected SC migration occurs upon antibody-mediated neutralization of Ctgf

Peripheral nerves are able to regenerate in response to injury; however, the molecular events underlying this process remain to be fully resolved. To further our understanding into this crucial mechanism, we employed a range of in vitro and in vivo approaches that identified key roles for H_2_O_2_ and Ctgf in nerve regeneration. We found that in response to axon injury, mitochondria rapidly generate H_2_O_2_ to activate surrounding SCs. The local synthesis and release of H_2_O_2_ induce extensive transcriptional changes at the injured NMJ, particularly in genes encoding for ECM components, including the YAP-dependent *Ctgf* up-regulation. Ctgf contributes to functional recovery upon proximal and distal nerve damage by assisting SC migration and, in turn, allowing the oriented re-growth of the axons. The major findings of the present study are summarized in Fig. 10.

Regeneration of the NMJ is orchestrated by several signaling pathways that control synaptic plasticity and remodeling upon injury [1, 6, 10]. To dissect the molecular determinants driving NMJ regeneration, we took advantage of a highly specific pre-synaptic neurotoxin as a model of acute and reversible degeneration of the nerve terminal, and profiled the transcriptional changes taking place at the murine NMJ during intoxication, revealing a striking ECM remodeling triggered by nerve injury. To address whether this process is triggered by H_2_O_2_, a major SC activator and a pro-regenerative factor [20, 22, 23], we cross-referenced the transcriptomic changes identified at α-LTx poisoned NMJs with those previously obtained from H_2_O_2_-treated primary SCs [23], identifying 169 common DEGs. Several of the 85 common up-regulated transcripts encode for ECM components (both structural and signaling members), among them the matricellular protein Ctgf. Matricellular proteins do not participate in ECM structural integrity, but rather act as modulators in a variety of cellular responses [25]. They are highly expressed during development or upon injury and, once secreted and sequestered in the extracellular space, they bind to the structural matrix and transduce signalling cascades, or contribute to activation of cytokines, growth factors, and proteases in the pericellular space. They also display de-adhesive activities, which likely contribute to tissue remodelling following injury, or in disease states. Indeed, matricellular proteins are involved in highly dynamic processes like wound healing, cancer, and in the production of connective tissue [52]. As the *Ctgf* transcript is part of the ‘H_2_O_2_ signature’, is increased during MAT degeneration and recovery, encodes for a matricellular protein involved in tissue remodelling, and is required for zebrafish spinal cord regeneration [53], we investigated for the first time in mice the cellular origin and the molecular determinants of Ctgf production, and its role in peripheral nerve regeneration.

At the intoxicated NMJ, the expression of *Ctgf* mRNA strongly increases during the acute phase of degeneration, with a second peak during regeneration. Ctgf localizes in PSCs and in the extracellular space, and promotes the functional recovery of the NMJ, as Ctgf neutralization by a specific antibody delays MAT regeneration following the acute injury by α-LTx. Arguably, *Ctgf* expression is triggered by *alarmins* from degenerating MAT: indeed H_2_O_2_, a major neuronal *alarm* signal, and a strong mediator of NMJ rescue, promotes Ctgf synthesis and release by primary SCs. Overall, injury signals released by damaged MNs induce *Ctgf* expression by SCs.

A novel and important finding reported here is that H_2_O_2_ is rapidly generated in the sciatic nerve upon damages that mimic traumatic injuries in humans (e.g., compression or transection), and this inter-cellular signaling drives *Ctgf* expression. Pro-regenerative axonal reactive oxygen species (ROS) signaling was recently found to occur in sensory neurons within a few hours from sciatic nerve crush, secondary to inflammatory signals coming from the inflammatory milieu [54]. It should be considered that the term ROS includes unstable and short-living species, such as the superoxide anion, which are unlikely to mediate any direct signaling given their rapid quenching. By converting superoxide anions into H_2_O_2_, the superoxide dismutase produces a more stable ROS species that can perform transfer from one cell to another, which is an absolute requirement for an inter-cellular mediator. Here, we not only identified H_2_O_2_ as the ROS species produced upon injury in the sciatic nerve of live, anaesthetized mice, but also found that it is generated by the mitochondria of injured nerves. Strikingly, this localized H_2_O_2_ signal is detectable within a couple of minutes from nerve injury.

H_2_O_2_ triggers Ctgf generation close to the injury site mainly in SCs, and particularly in the *bridge*, a new tissue that forms upon nerve transection to reconnect the two stumps. Of note, *bridge* SCs are a peculiar sub-population of de-differentiated SCs that display pronounced mesenchymal features (in response to the microenvironment) and migratory behavior during nerve repair [46].

Ctgf consists of four functional domains that mediate its participation in several physiological actions. The N- and C-domains of Ctgf mediate distinct biological processes, *i.e.*, differentiation (collagen synthesis), and proliferation, respectively, in concert with other growth factors [55]. Pro-fibrogenic properties of Ctgf are commonly ascribed to the N-terminus, and have been widely described in different pathological contexts. A human monoclonal antibody (FG-3019) raised against the N-domain of the molecule, is currently under evaluation in clinical studies for the treatment of idiopathic pulmonary fibrosis, pancreatic cancer, and Duchenne muscular dystrophy (DMD). In gastrocnemius muscles of SOD1^G93A^ mice at symptomatic stages (16 weeks), which show increased levels of Ctgf, administration of FG-3019 reduced fibrosis and ameliorated NMJ innervation [56]. Ctgf is up-regulated in skeletal muscles of *mdx* mice modelling DMD, and its genetic reduction in this model through crossing with heterozygous CTGF^±^ mice leads to improved skeletal muscle isometric force, and decreased muscle fibrosis and damage [57]. In addition, in mice heterozygous for Ctgf, or upon FG-3019 treatment, there is an accumulation of ECM proteins after denervation, as compared to control mice, suggesting a direct role of Ctgf in denervation-induced fibrosis [58].

Conversely, very little is known about the pro-regenerative potential of Ctgf. One study in zebrafish [53] reports that Ctgf*a* supports glia proliferation and bridging, in turn promoting spinal cord regeneration. Our data support this notion of the pro-regenerative properties of Ctgf: these are likely ascribable to the C-terminus of the molecule, which is specifically targeted by the anti-Ctgf antibodies employed in our neutralization experiments.

Growing evidence supports a crucial role of the cellular milieu for the regeneration response [46], which likely influences Ctgf action; in fact, Ctgf may stimulate either cell proliferation or cell differentiation, depending on the presence or absence of additional growth factors and cytokines in the milieu. Accordingly, in tissue formation and in regeneration both processes occur, with cell proliferation preceding and then closely followed by differentiation [55]. Both zebrafish [53] and our mouse injury experimental models are competent for regeneration, at variance from that employed by Rebolledo and colleagues, where a small section of the nerve was removed after unilateral denervation to prevent re-innervation, making regeneration unfeasible [58]. Thus, the pro-regenerative context of the two models seems to be crucial to determine the exact role of Ctgf in regeneration.

Ctgf sustains nerve regeneration most likely via an axon growth promoting action. Indeed, in the absence of Ctgf, axonal re-growth is slower and appears less polarized, likely due to a slower and misdirected migration of SCs within the bridge, eventually leading to a delay in neurotransmission rescue. Of note, the *Ctgf* transcript increases in neurons responsible for collateral sprouting, which is a process where undamaged neurons react to an injury-induced environment (Wallerian degeneration) by expanding sprouts that functionally synapse with denervated targets [59]. Along the same line, Ctgf-positive sprouts, which form in PSCs upon neurotransmission blockade (by either denervation, or intoxication), further support the view that SC-expressed Ctgf may play a role in supporting/guiding axonal elongation.

*Ctgf* expression may also rely on mechanical constraints, e.g., changes in the stiffness of ECM and/or loss of axonal contact caused by nerve damage. Indeed, context-dependent signalling can be sensed by SCs through mechanosensors and mechanotransducers [60–62] and, in turn, affect *Ctgf* gene expression. Accumulating evidence has described pivotal functions of the Hippo pathway in regulation of cell plasticity during mammalian development and tissue regeneration [63]: here we show that the transcriptional coactivator YAP, a major downstream effector of the Hippo pathway, drives *Ctgf* transcription in response to a local inter-cellular H_2_O_2_ signalling, paving the way for a future further investigation on the role of YAP-mediated mechanotransduction in peripheral nerve repair and in SC biology, and on the contribution of local H_2_O_2_ in such communication.

The present findings support the view that an intense inter-cellular signalling, of which H_2_O_2_ is a major player, occurs not only during development [7, 9], but also in adulthood, and drives peripheral nerve regeneration. Moreover, it highlights how our time-series transcriptional profiling of the regenerating NMJ represents a powerful source of novel candidate molecules that can be harnessed to support regeneration in different peripheral nerve injuries and possibly neuropathies.

## Supplementary Information

**Additional file 1**. Supplementary figures.

**Additional file 2: Table S1.** Differential analyses of gene expression at the α-LTx-poisoned soleus NMJs during degeneration and regeneration Differential analyses comparing gene expression at the α-LTx-poisoned soleus NMJs at the four time points analyzed with respect to controls. Log2-fold change, p-value and class (- down-regulated, + up-regulated, = invariant), and the average count of reads per million (CPM) are reported for each gene and each time point.

**Additional file 3: Table S2.** Gene ontology and REACTOME analysis of α-LTx data set. Gene ontology and REACTOME pathways enrichment analysis of DEGs of the α-LTx data set (down- and up-regulated) at each time point. The number and the list of DEGs associated to significant terms are reported.

**Additional file 4: Table S3.** Intersection of α-LTx and H2O2 data sets.

**Additional file 5: Table S4.** Gene ontology and REACTOME analysis of α-LTx-H2O2 common DEGs Intersection of DEGs of the α-LTx data set with those induced by H2O2, previously identified in [23]. The class (- down-regulated, + up-regulated, = invariant) of each gene of the two data sets is reported.

**Additional file 6: Table S5.** List of up-regulated ECM terms shared by the α-LTx and the H2O2 data sets. Selection of GO and REACTOME terms associated to the ECM emerging from the intersection of up-regulated genes of the α-LTx and the H2O2 data sets.

**Additional file 7: Movie 1.** 3D movie showing Ctgf localization in a control soleus NMJ.

**Additional file 8: Movie 2** 3D movie showing Ctgf localization in a degenerated soleus NMJ.

**Additional file 9: Table 6.** List of antibodies employed in the study and relative information.

## Data Availability

Raw and analyzed RNA-seq data generated in this study have been deposited in the Gene Expression Omnibus (GEO) under the accession code GSE154547. Part of the data are included as electronic supplementary material.

## Abbreviations

BoNT/A: Botulinum neurotoxin type A
ChAT: Choline acetyltranferase
Ctgf: Connective tissue growth factor
DEG: Differentially expressed genes
GAP-43: Growth-associated protein 43
H_2_O_2_: Hydrogen peroxide
α-Ltx: α-Latrotoxin
MAT: Motor axon terminal
MN: Motor neuron
NF: Neurofilament
NMJ: Neuromuscular junction
NOX: NADPH oxidase
PSC: Perisynaptic Schwann cell
ROS: Reactive oxygen species
SC: Schwann cell
SCMN: Spinal cord motor neuron
SNAP-25: Synaptosomal-associated protein, 25 kDa
TEAD: TEA domain family member 4
VAMP: Vesicle associated membrane protein
YAP: Yes-associated protein
