# Supplementary material for: Hydrogen peroxide induced by nerve injury promotes axon regeneration via connective tissue growth factor
Source: Acta Neuropathol Commun. 2022 Dec 25;10:189. doi: 10.1186/s40478-022-01495-5 (PMC9791753; doi:10.1186/s40478-022-01495-5)
Supplement: Supplementary file 1 — Additional file 1. Supplementary figures. [file 40478_2022_1495_MOESM1_ESM.docx]

**Additional file 1**

Hydrogen peroxide induced by nerve injury promotes axon regeneration via Connective Tissue Growth Factor

Samuele Negro, Fabio Lauria, Marco Stazi, Toma Tebaldi, Giorgia D’Este, Marco Pirazzini, Aram Megighian, Francesca Lessi, Chiara M Mazzanti, Gabriele Sales, Chiara Romualdi, Silvia Fillo, Florigio Lista, James N. Sleigh, Andrew P. Tosolini, Giampietro Schiavo, Gabriella Viero and Michela Rigoni^*^

*Correspondence to: [michela.rigoni@unipd.it](mailto:michela.rigoni@unipd.it)

Additional file 1 includes 6 Supplemental Figures

**
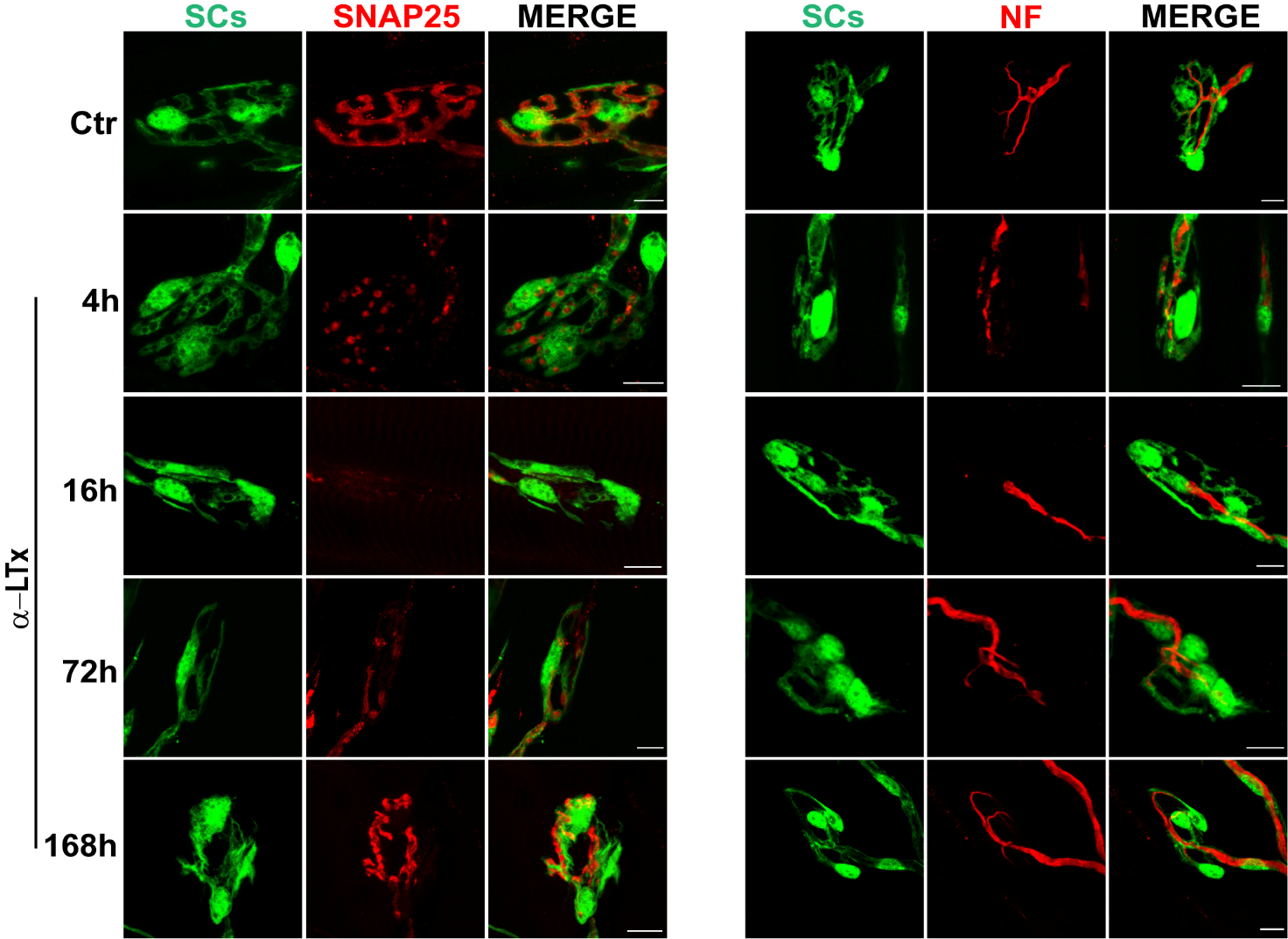
**

**Fig. 1** Time-course of MAT degeneration and regeneration at soleus NMJs following α-LTx treatment. Progressive SNAP25 and NF (*red*) disappearance and reappearance at soleus NMJs of *plp*-GFP mice (expressing GFP in SCs) during the reversible MAT degeneration induced by α-LTx. PSCs are GFP-positive (*green*). Scale bars: 10 µm.

**
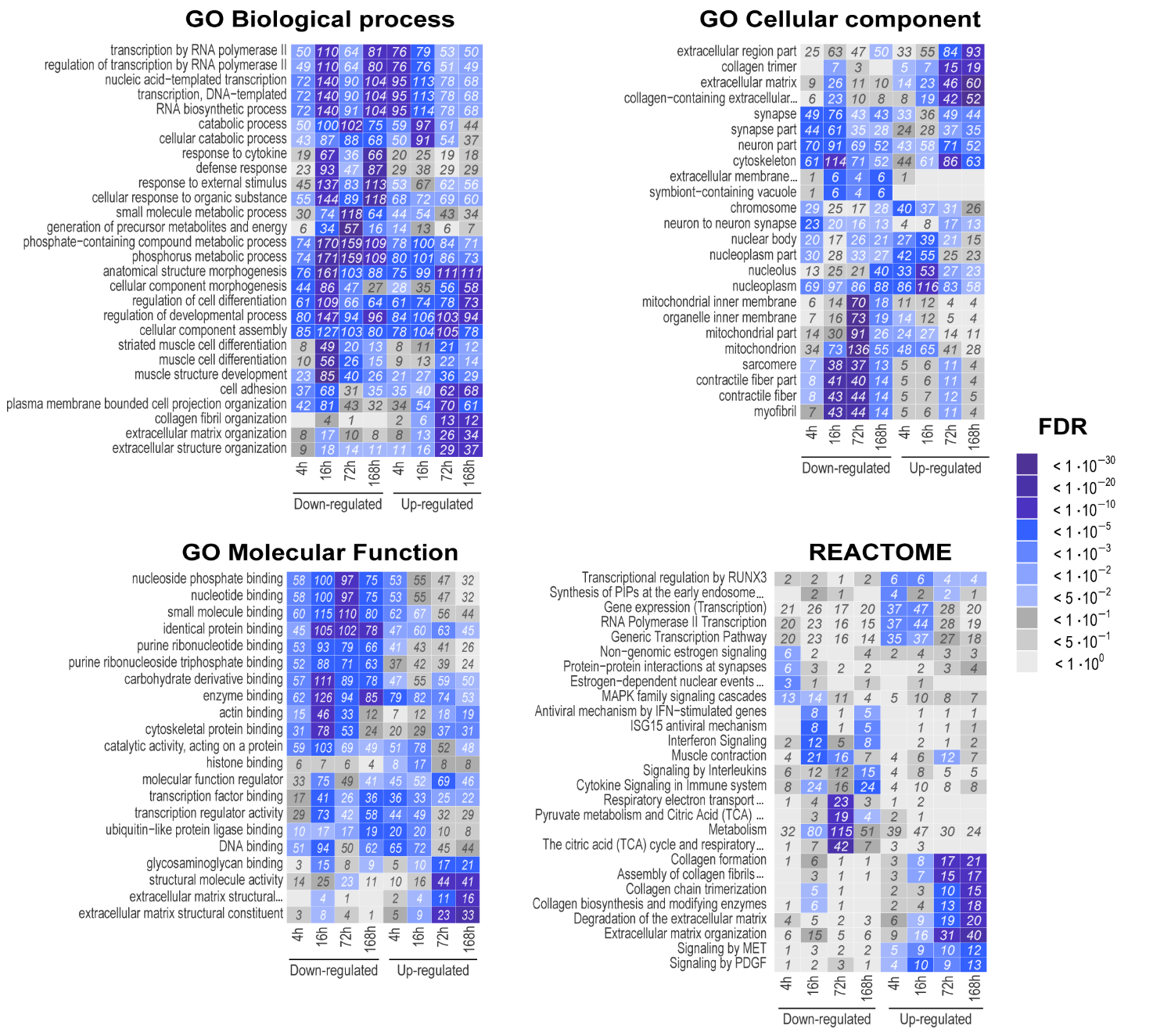
**

**Fig. 2** Gene ontology of the NMJ data set. Enriched functional terms from Gene Ontology (cellular component, biological process and molecular function) and REACTOME pathways enrichment analysis, performed on down- and up-regulated DEGs at each time point. Cells are filled according to FDR values, and display the number of genes associated to the corresponding term.


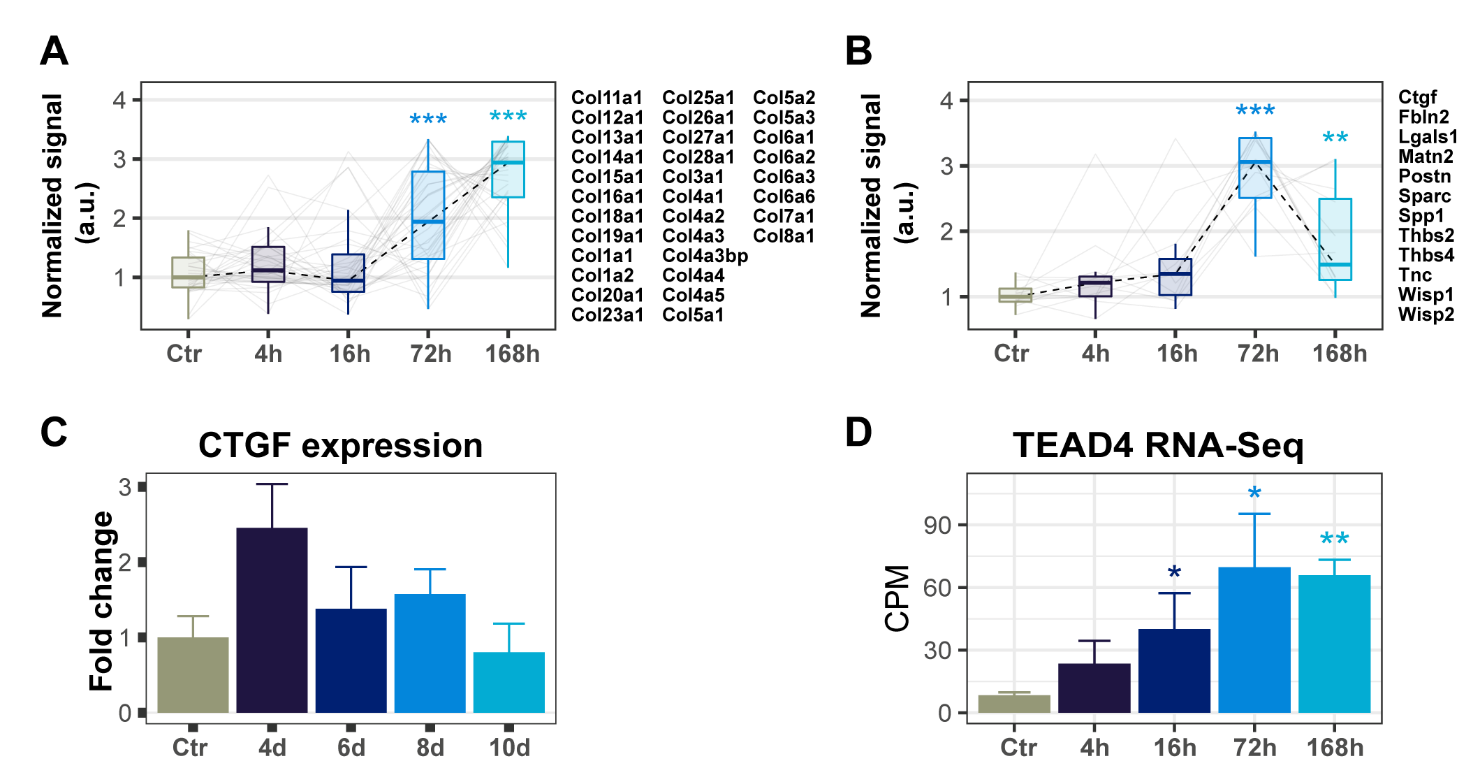


**Fig. 3** The expression of ECM components increases during regeneration. **A, B** Expression trajectories of DEGs encoding different collagen isoforms (**A**) and regulatory ECM proteins (**B**). Each plot represents the expression trajectories of the single genes, together with the median (dotted line) and summary statistics (boxplot). The genes included in the analysis are listed on the right of each plot. Significant changes during the time-course of α-LTx treatment with respect to the control were determined using a Wilcoxon-Mann-Whitney test. ***p<0,001 (**A**), ***p<0,001 and **p=0.0018 (**B**), a.u.=arbitrary units. **C** *Ctgf* mRNA levels (expressed as fold change with respect to the control) in bridge SCs during nerve regeneration post-sciatic nerve transection (adapted from *Clements et al, 2017*). **D** RNA seq analysis showing the mRNA levels of the transcription factor TEAD4 at the NMJ during the time course of α-LTx treatment, normalized to the control. The reported significant differences were determined by differential expression analysis. N=5, *p=0.0348 (16 h), *p=0.0109 (72 h), **p=0.0087 (168 h).


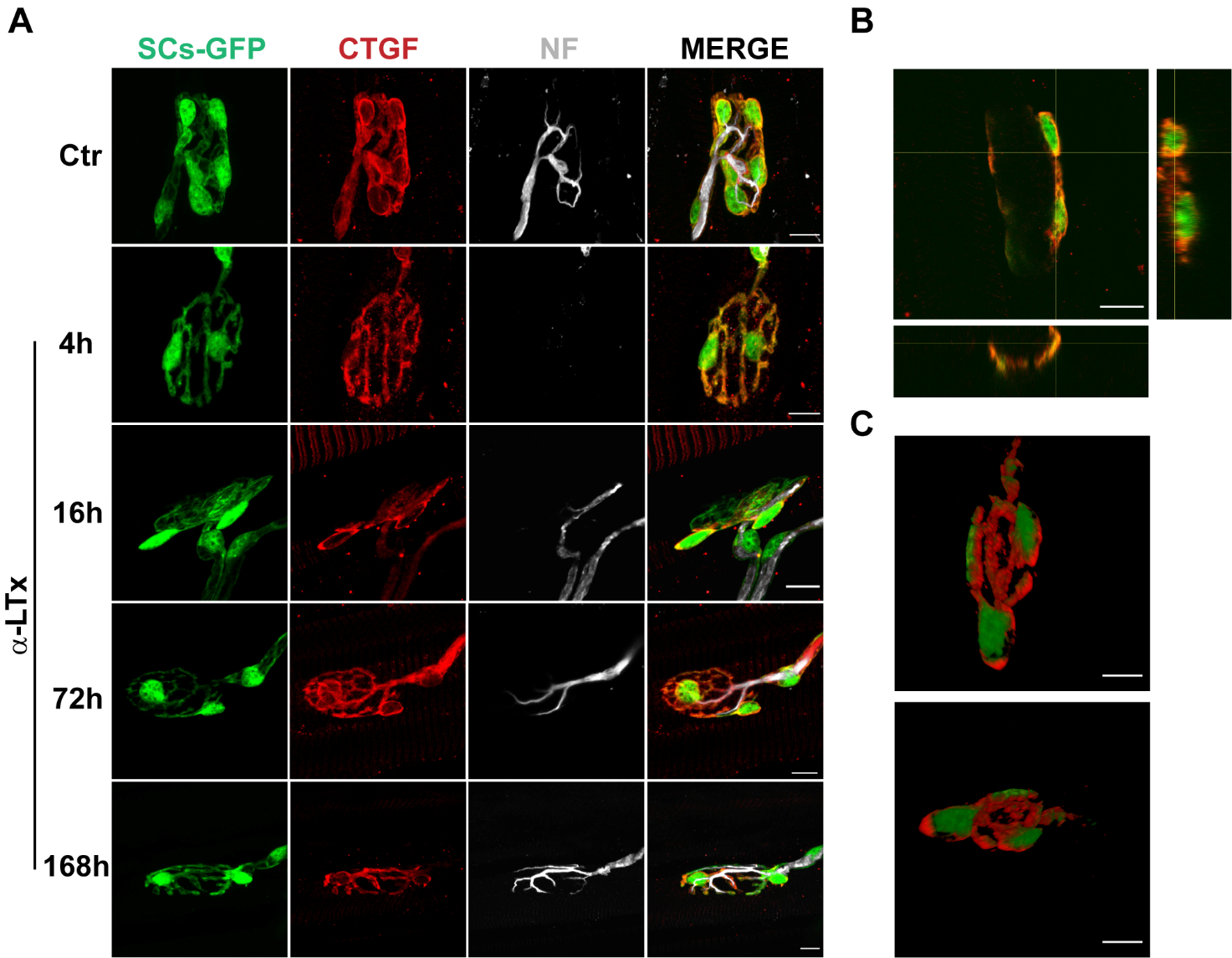


**Fig. 4** Ctgf localizes to the NMJ and redistributes upon toxin-induced injury. **A** Time-course of Ctgf (*red*) expression at LAL NMJs during intoxication by α-LTx (4, 16, 72 and 168 h). Control NMJs are in upper panels. PSCs are GFP-positive (*green*) and the neuronal-component neurofilament-positive (NF, *white*). Scale bars: 10 µm. **B** Orthogonal projection showing Ctgf localization in PSCs and in the ECM at a control NMJ. Scale bar: 10 µm. **C** 3D reconstruction of a control NMJ. Scale bars: 10 µm.

**
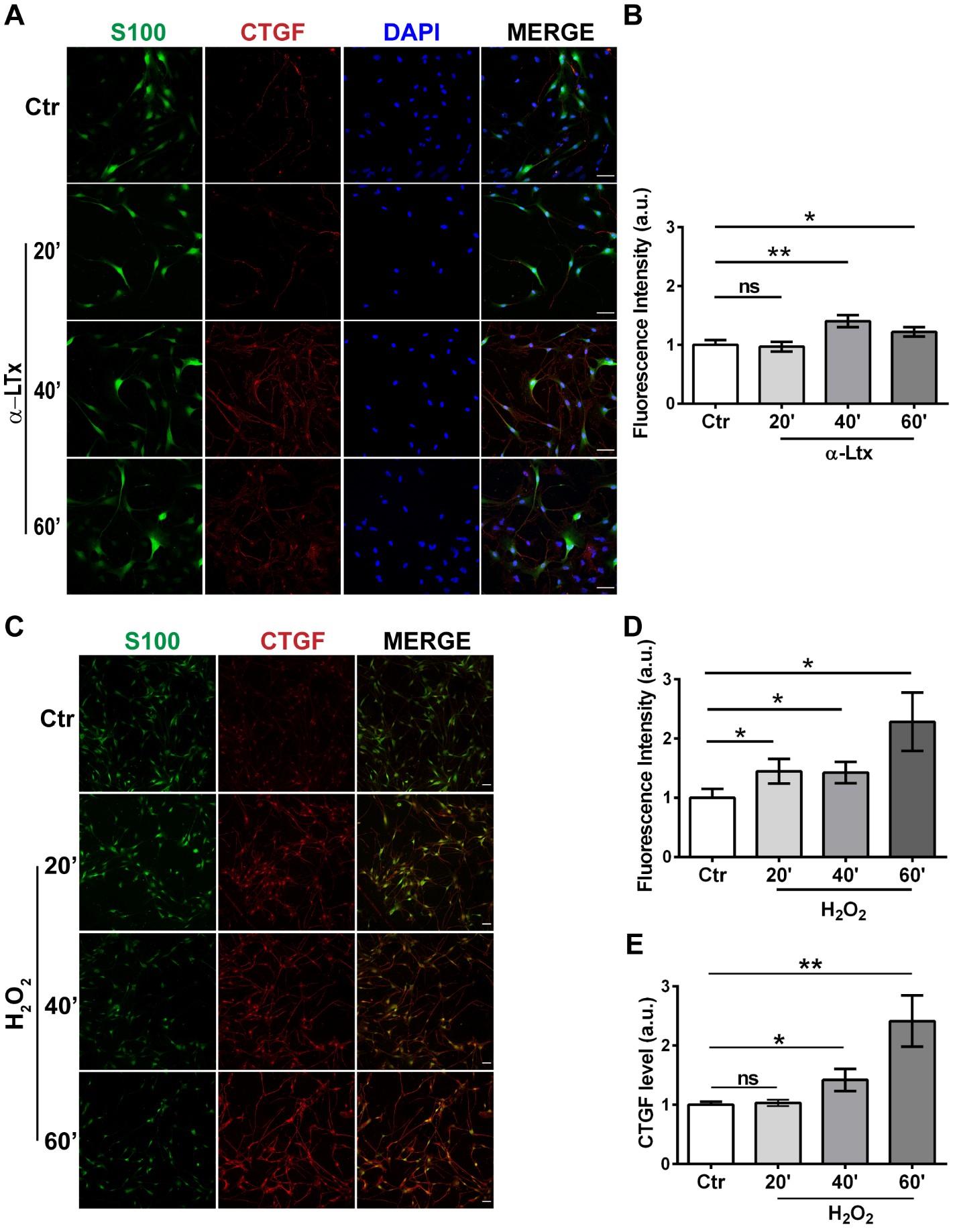
**

**Fig. 5** Hydrogen peroxide induces Ctgf expression and release in primary SCs. **A, B** SCMN injury triggers Ctgf expression in co-cultured SCs. Co-cultures of primary SCMNs and primary SCs were exposed to α-LTx for 20, 40 and 60 min, and stained for Ctgf (*red*) and S100 (*green*) to identify SCs. Nuclei are in *blue* (**A**). Scale bars: 50 µm. Quantification of Ctgf signal is shown in **B**. N=3, 120 cells analyzed. *p=0.028, **p=0.006, ns= not significant, a.u.=arbitrary units. **C, D** Immunostaining for Ctgf (*red*) of primary SC cultures (S100 positive, *green*) exposed to 50 µM H_2_O_2_ for 20, 40 and 60 min, and relative quantitation (**D**). Scale bars: 50 µm (**C**). (**D**) N=3, 120 cells analyzed. *p=0.039 (20 min), *p=0.034 (40 min), and *p=0.012 (60 min), a.u.=arbitrary units. **E** Quantification of Ctgf release upon exposure of primary SCs to 50 µM H_2_O_2_ for 20, 40 and 60 min by ELISA. N=4, *p=0.020, **p=0.0049, ns=not significant, a.u.=arbitrary units


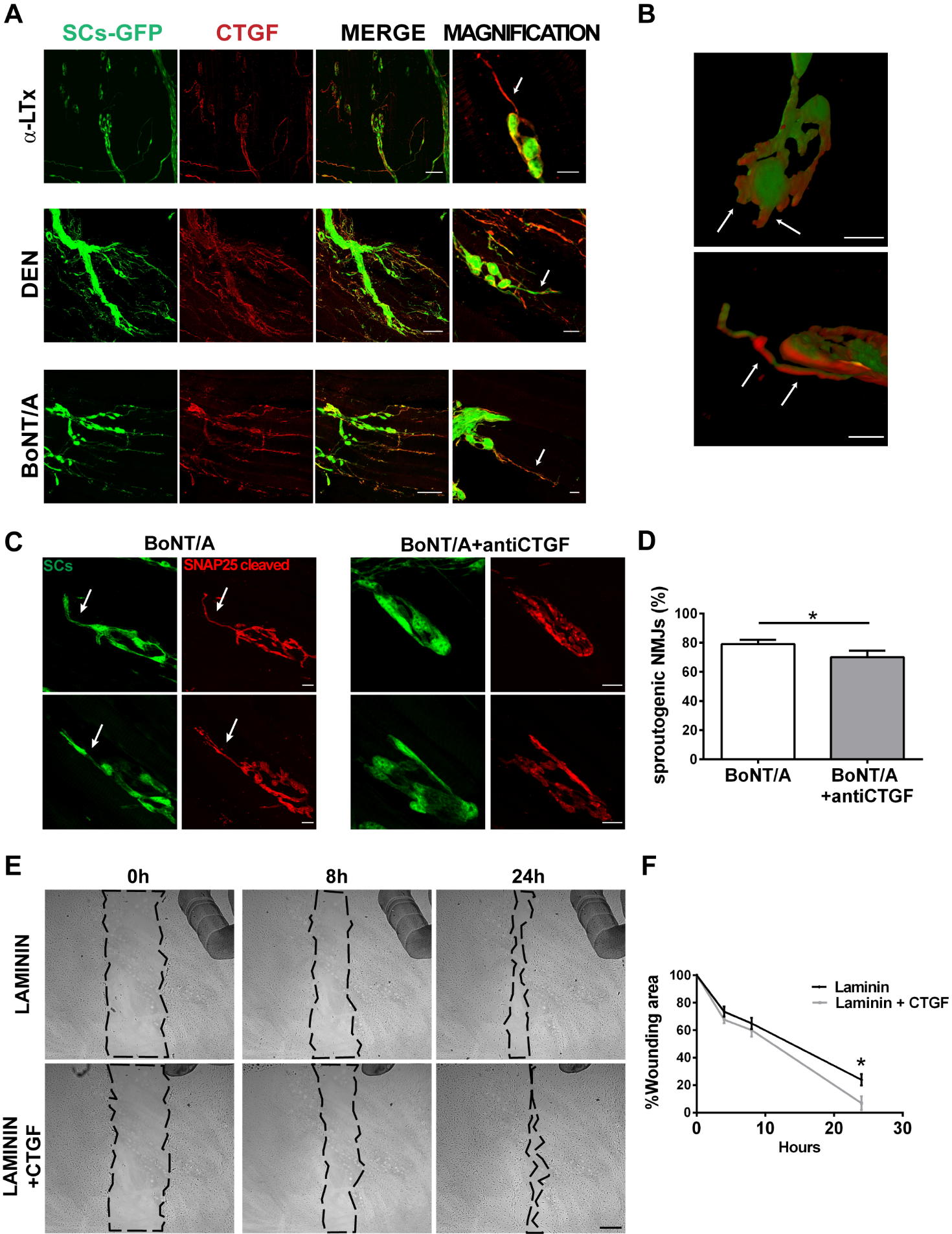


**Fig. 6** Glial sprouts express Ctgf to support SC migration. **A** Ctgf-positive (*red*) PSC sprouts (arrows) in LAL muscles 16 h after α-LTx injection (upper panels), 21 days after sciatic nerve transection (middle panels), and 21 days upon BoNT/A intoxication (lower panels), and the correspondent magnifications. PSCs are in *green*. Scale bars: 50 µm (10 µm in the magnifications). **B** 3D reconstructions of NMJs 4 h (upper panel) and 16 h (lower panel) from α-LTx poisoning. Scale bars: 10 µm. PSCs are in *green*, Ctgf in *red*. Arrows point to Ctgf-positive sprouts. **C, D** Ctgf neutralization affects PSC sprouting at soleus NMJs after chemical denervation by BoNT/A. PSC (*green*) sprouts (*arrows*) form upon neurotransmission blockade induced by BoNT/A (**C**, left panels). BoNT/A proteolytic activity is revealed by an antibody recognizing the cleaved form of SNAP25 (*red*). BoNT/A-induced sprouting is reduced when Ctgf is neutralized (**C**, right panels). Scale bars: 10 µm. Relative quantitation is shown in **D**. N=5. One hundred NMJs analyzed/muscle. *p=0.043.  **E, F** Wound healing assay showing the time-course of SC migration *in vitro* following scratching (performed at time 0). Cells were plated on a laminin (upper panels) or laminin + recombinant Ctgf (lower panels) coating. Scale bars: 50 µm. Quantification of the wound area at different time points from scratching is shown in **F**. N=3, *p=0.0105
