# Supplementary material for: Hydrogen peroxide induced by nerve injury promotes axon regeneration via connective tissue growth factor
Source: Acta Neuropathol Commun. 2022 Dec 25;10:189. doi: 10.1186/s40478-022-01495-5 (PMC9791753; doi:10.1186/s40478-022-01495-5)
Supplement: Supplementary file 9 — Additional file 9: Table 6. List of antibodies employed in the study and relative information. [file 40478_2022_1495_MOESM9_ESM.pdf]

Antibodies	Dilution	SOURCE
Rabbit polyclonal anti-Ctgf.<br><br>CTGF is a matricellular protein. It is involved in different biological processes, such as cell proliferation, angiogenesis, wound healing and tissue fibrosis.	1:200	Abcam Cat# ab6992; RRID:AB_305688
Rabbit polyclonal anti-Ctgf.<br><br>CTGF is a matricellular protein. It is involved in different biological processes, such as cell proliferation, angiogenesis, wound healing and tisse fibrosis.	2µg/40µl	Fitzgerald Cat # 70R-CR023; RRID:AB_10815875
Alexa Fluor® 555 alpha-bungarotoxin.<br><br>Alpha-bungarotoxin a 74-amino acid peptide that binds with high affinity to the α-subunit of the nicotinic acetylcholine receptor (AChR) of neuromuscolar junctions	1:200	Thermofisher Cat# B35451, RRID:AB_2617152
Rabbit polyclonal anti-S100-A1.<br><br>S100 proteins are small EF-hand calcium binding proteins. S100 is a Schwann cell marker	1:400	Agilent Cat# Z0311, RRID:AB_10013383
Rabbit monoclonal anti-GAP43<br><br>Growth-associated protein 43 is an intracellular protein located at branching points, growth cones, and axon terminals involved. It is a marker of axonal and synaptic growth.	1:200	Abcam Cat# ab75810, RRID:AB_1310252
Chicken polyclonal anti-NF.<br><br>Neurofilaments are intermediate filaments that comprise the neuronal cytoskeleton.	1:800	Abcam Cat# ab4680, RRID:AB_304560
Rabbit polyclonal anti-VAMP1<br><br>Vesicle associated membrane protein 1 is aprotein involved in the molecular regulation of transmitter release at the presynaptic nerve terminal	1:100	Rossetto et al,1996
Mouse monoclonal anti-SNAP25<br><br>Synaptosomal associated protein 25 is a presynaptic protein critical for neuroexocytosis	1:200	Abcam Cat# ab24737, RRID:AB_448273
Rabbit polyclonal anti-SNAP25 BoNT/A-cleaved<br><br>Antibody specific for BoNT/A cleaved form of SNAP25	1:200	Antonucci et al, 2008

Rabbit polyclonal anti-syntaxin 1A/1B<br><br>Syntaxin1 is a plasma membrane protein essential for synaptic vesicle fusion	1:100	Zanetti et al,2017
Rabbit monoclonal anti-YAP<br><br>YAP (Yes-associated protein, YAP65) is a transcriptional coactivator protein acting via binding to the TEAD transcription factor	1:200	Cell Signaling Cat#14074<br>RRID:AB_2650491
Secondary AlexaFluor antibodies 488	1:200	Thermo Fisher
